# Characterization of degradation and heterozygote balance by simulation of the forensic DNA analysis process

**DOI:** 10.1007/s00414-016-1453-x

**Published:** 2016-11-03

**Authors:** Oskar Hansson, Thore Egeland, Peter Gill

**Affiliations:** 1 Norwegian Institute of Public Health, Department of Forensic Biology, Oslo, Norway; 2 Faculty of Medicine, University of Oslo, Oslo, Norway; 3 Norwegian University of Life Sciences, Ås, Norway

**Keywords:** PCR, Heterozygote balance, Simulation, Degradation, Validation

## Abstract

Simulation experiments were used to show the impact of varying extraction efficiency, aliquot proportion, and PCR efficiency on the heterozygote balance of a range of diploid and haploid cells. Reducing either parameters introduces variance. It is well-known that the variance in heterozygote balance increases as the amount of DNA is reduced. Surprisingly the distribution is in fact diamond shaped — the variance start to decrease at very low amounts of DNA. Simulations suggest that pristine diluted DNA is an acceptable approximation in validations to infer heterozygote balance. However, the difference in distribution of the variance between diploid and haploid cell types may, under some circumstances, need to be considered in statistical models. Finally, we exemplify how simulations can be used to predict the outcome of PCR for degraded samples. Visualizing the predicted DNA profile as an electropherogram can help to identify the best approach for sample processing.

**Electronic supplementary material:**

The online version of this article (doi:10.1007/s00414-016-1453-x) contains supplementary material, which is available to authorized users.

## Introduction

The typical forensic DNA analysis process consists of sample recovery, extraction, quantification, amplification, and capillary electrophoresis. Depending on the laboratory’s instrumentation and workflow, there can be additional purification of the DNA extract or PCR product. Forensic laboratories seek to optimize each step of the process to maximize the chance to retrieve DNA evidence. Interpretation of DNA evidence within the likelihood ratio framework has made significant progress in recent years [1, 2] and several software solutions are available [3–5]. Different models, e.g. qualitative, semi-continuous (without peak height), and continuous (with peak height, stutter, etc), have been implemented. The only assumptions of the qualitative model are the estimated allele frequencies. Usually, unrelatedness between the contributors to the DNA evidence is also assumed, although recently, it has been possible to specify relatedness in some software (e.g. LRmix Studio [4]). Semi-continuous models include drop-in and drop-out parameters, estimated either from laboratory experiments or from the observed DNA evidence and simulations. Continuous models (with peak height) may require additional parameters to be estimated experimentally, although this is largely circumvented by gamma models [6] which automatically determine parameters from the crime stain profile itself. Typically a number of known single-source samples are analysed to estimate key characteristics like stutter ratios and heterozygote balance. Each extraction method, STR kit, PCR protocol, and capillary electrophoresis (CE) instrument may result in different estimates of the parameters. To ensure validity, the experiments may need to be repeated if there are significant changes to protocols or equipment (e.g. major service of the CE instrument).

In the laboratory, there is always a loss of DNA during the DNA extraction process [7–9]. Although there is limited published information on the absolute efficiency, NIST studies indicate an absolute DNA extraction efficiency of 1–37 %.^1^

The PCR process is not 100 % efficient, [10] estimated the PCR efficiency to be approximately 82–97 % using real-time PCR. Gill et al. [11] created a graphical simulation model of the entire DNA process using a binomial selection of molecules, as suggested by [12], in order to simulate each step of the process. The model was used to predict the behaviour of heterozygote balance and the probability of allelic drop-out for low-template samples.

This paper explores the effect of different parameters (such as PCR efficiency and aliquot) on the heterozygote balance for diploid and haploid cells. Diploid cells contain two copies of each chromosome, while haploid cells (i.e. sperm cells) contain only one copy. Simulation was used to predict the stochastic behaviour of sub-cell (pg) levels of DNA, which was compared to observed experimental data. Finally, implications to casework relative to assumptions used in continuous models are discussed.

## Material, methods, and models

### Parameters and definitions

- Extraction efficiency (*e* *x*_*e*_): The probability that a given DNA molecule survives the DNA extraction process.
- PCR aliquot (*p* *c* *r*_*a*_): A proportion of the DNA extract is transferred to the PCR tube. Therefore, there is a probability that a given DNA molecule will be selected for PCR amplification.
- PCR cycles (*p* *c* *r*_*c*_): Number of PCR cycles.
- PCR efficiency (*p* *c* *r*_*e*_): During each PCR cycle, there is a probability that a given DNA molecule will be amplified.
- Stutter probability (*s* *t* *u* *t* *t* *e* *r*_*p*_): During each PCR cycle, there is a probability that a given DNA molecule will be amplified as a stutter one repeat shorter than the allele.
- Aliquot to capillary electrophoresis (*c* *e*_*a*_): A proportion of the PCR product is transferred to the capillary electrophoresis injection plate.
- Capillary electrophoresis peak height threshold (*c* *e*_*T*_): The number of fluorescent labelled DNA molecules required to trigger a signal described by the intercept (*c* *e*_*Ti*_), slope (*c* *e*_*Ts*_), and the residual standard error (*c* *e*_*Tσ*_).
- Capillary electrophoresis peak height scaling (*c* *e*_*S*_): Conversion of the number of DNA molecules into relative fluorescent molecules (RFU) described by the intercept (*c* *e*_*Si*_) and slope (*c* *e*_*Ss*_).
- Limit of detection threshold (*LDT*): Signals above this peak height threshold (RFU) is considered to be reliably caused by actual alleles rather than instrument noise.
- Degradation parameter (*P*(*d* *e* *g*)): The probability of degradation per base pair. If a DNA fragment is degraded at one or more bases the amplification of that fragment fails.
- Degradation index (*DI*): The ratio of the low molecular weight target to the high molecular weight target provides a qualitative measure of the degradation.

### Simulated data

Simulation was performed using the R package *pcrsim* 2 version 1.0. The package was developed based on the simulation functions in *forensim* [13]. Both packages are implementations of ‘A graphical simulation model of the entire DNA process’ [11]. In *pcrsim* the PCR efficiency is assumed to be constant across cycle number, which has previously been demonstrated to be true for the first 10 to 15 cycles [12, 14]. In reality PCR efficiency declines towards the plateau phase mainly because of product inhibition of the DNA polymerase enzyme [15]. However, for STR analysis of low-template samples, the plateau phase is in practice never reached [16]. Hedell et al. [16] showed that for each increase in number of PCR cycles from 30 to 35, the allele peak height increase was approximately constant, coinciding with ideal amplification. Hence, the application of a constant PCR efficiency per cycle is a realistic approximation. Some published values of the PCR efficiency are 0.82 [11], 0.85 [17], and 0.82–0.97 [10]. We will use a PCR efficiency *p* *c* *r*_*e*_=0.90 to simulate crime stains. Specific simulation parameters are given under the respective simulation experiment. If direct PCR was used, then the extraction efficiency *e* *x*_*e*_=1.00 and the PCR aliquot *p* *c* *r*_*a*_=1.00 since none of the DNA is lost using this method.

In order to maximize the data collection for simulations with sub-cellular amounts of DNA, the capillary electrophoresis peak height threshold was set to *c* *e*_*T*_=0 with a peak height scaling of *c* *e*_*S*_=1. Consequently, there is no drop-out dependant on low peak height, only due to complete absence of template molecules in the PCR reaction.

#### Serial dilutions vs. crime stains

Parameters for comparison between simulated dilutions and simulated crime stains were as follows: the original experiment was a 2-fold serial dilution of a NIST human DNA quantitation standard (SRM 2372A) as outlined by [18]. In this simulation, the series was extended at the lower end with three more dilutions to produce a final range of 1.65 to 845 pg in the PCR reaction (equivalent to 0.25–128 diploid cells assuming 6.6 pg per cell). Stock solution of the quantitation standard was 57 ng/*μ*l and dilution was performed with large transfer volumes (450 *μ*l) to avoid stochastic effects. The comparison was made between 1000 replicate simulations of the same serial dilution, simulation of 1000 diploid crime stains with target 0.25-128 cells in the PCR reaction, and 1000 haploid crime stains with target 0.50–256 cells in the PCR reaction.

1. The extraction efficiency was set to *e* *x*_*e*_=0.30 (the higher end of previously reported values).^1^
2. The PCR aliquot was *p* *c* *r*_*a*_=0.35 (the higher end of commonly used proportions).^3^
3. A PCR efficiency of *p* *c* *r*_*e*_=0.90, approximately in the middle of a previously reported range of 0.82−0.97 [10], was used. The number of cycles was *p* *c* *r*_*c*_=28.
4. CE aliquot *c* *e*_*a*_=1.0.
5. CE detection threshold was set to *c* *e*_*Ti*_=14.03744, *c* *e*_*Ts*_=0.82254, *c* *e*_*Tσ*_=0.1319579 based on a previous 3500xL calibration.^4^
6. CE peak height scaling was set to: *c* *e*_*Si*_=−14.38233, *c* *e*_*Ss*_=1.173163 based on a previous 3500xL calibration.^4^
7. Limit of detection threshold *L* *D* *T*=200 RFU.

The crime stain samples were simulated using four settings (other parameters were the same as for the simulated dilution):

1. *e* *x*_*e*_=0.30 and *p* *c* *r*_*a*_=0.35, emulating a realistic process where a relatively efficient extraction method^1^ is combined with a relatively high aliquot proportion.^3^
2. *e* *x*_*e*_=0.30 and *p* *c* *r*_*a*_=1.00, emulating a relatively efficient extraction method^1^ combined with PCR of the entire DNA extract.
3. *e* *x*_*e*_=1.00 and *p* *c* *r*_*a*_=0.35, emulating single tube extraction combined with a relatively high aliquot proportion.^3^
4. *e* *x*_*e*_=1.00 and *p* *c* *r*_*a*_=1.00, emulating direct PCR.

#### Heterozygote balance and the ‘diamond’ effect

For comparison with observations from experimental data, simulation parameters were used that emulated the experimental conditions as closely as possible. For comparison to [16], the following parameters were used.

1. The extraction efficiency was set to *e* *x*_*e*_=1.00 to mimic one tube Chelex extraction.
2. The aliquot forwarded to PCR was set to *p* *c* *r*_*a*_=0.05.
3. For each simulated sample *p* *c* *r*_*c*_=30−35 PCR cycles with PCR efficiency *p* *c* *r*_*e*_=0.90, approximately in the middle of a previously reported range of 0.82−0.97 [10], was used.
4. Stutters were simulated by multinomial selection with *s* *t* *u* *t* *t* *e* *r*_*p*_=0.005 [19].
5. CE detection threshold was set to *c* *e*_*Ti*_=15.4653, *c* *e*_*Ts*_=0.9044, and *c* *e*_*Tσ*_=0.364 based on a previous 3130xL calibration.^4^
6. CE peak height scaling was set to *c* *e*_*Si*_=−13.66131 and *c* *e*_*Ss*_=1.0047, *c* *e*_*Sσ*_=0.3836 based on a previous 3130xL calibration.^4^
7. The limit of detection threshold was set to *L* *D* *T*=50 RFU.

Possible stutter-allele pairs, when the actual partner allele has dropped out, were excluded from the calculations by removing alleles separated with 1 repeat unit. Loci with mean peak heights >10,000 RFU were removed to mimic the saturation threshold of the 3130xL instrument.

#### Degraded samples

See on-line supplement Section C for details of simulation parameters for degraded samples.

### Empirical data

#### Heterozygote balance

Empirical data used by [16] was kindly provided by the authors. Their experimental set-up was as follows. A dilution series was prepared, by mixing 5 *μ*l whole blood together with 1245 *μ*l of 0.9 % NaCl (commonly referred to as physiological saline). The volume of diluted blood transferred in each subsequent step was at least 400 *μ*l to avoid potential stochastic effects. The use of physiological saline prevented cell lysis; hence, the integrity of complete genomes was conserved. Quantification was performed in triplicate using the Quantifiler®; Human DNA Quantification Kit (Life Technologies). Only three out of twelve samples produced results within the range of the standard curve. Two were negative and the remaining were extrapolated from the standard curve. Therefore [16] estimated the concentrations for the three lower concentrations based on the sample with the highest concentration. See reference [16] for further details on the experimental set-up. The actual quantification results are reproduced in Table 4 (online supplement Section A).

#### Degraded samples

Anonymous DNA extracts from nine presumably degraded tissue samples were used. The extraction method was the BioRobot EZ1 (Qiagen) using the EZ1 DNA Tissue Kit (Qiagen) according to manufacturers recommendations. The extracts had been stored for about two years in a freezer prior to quantification and analysis. The Quantifiler®; Trio DNA Quantification Kit (Applied Biosystems), with an 80 bp small autosomal target and 214 bp large autosomal target, and PowerQuant^TM^ System (Promega), with an 84 bp small autosomal target and 294 bp large autosomal target, were used for quantification. Both kits confirmed that the tissue samples were degraded to different degrees (degradation index in Table 2). The PowerPlex®; ESX 17 Fast System (Promega) was used for STR amplification.

### Analysis of data

The free R statistical software^5^. was used to analyse data. Specifically the package *strvalidator* [20] version 1.4 was used to calculate heterozygote balance according to Eq. 1:

1 $$ Hb=\frac{{\O}_{HMW}}{{\O}_{LMW}}  $$

where *H* *b* is the heterozygote balance, *Ø*_*HMW*_ and *Ø*_*LMW*_ are the simulated number of amplicons (if no scaling is used) and simulated peak height (if scaling is used) of the high and low molecular weight allele, respectively.

The R packages *data.table* 6. and *plyr* 7. were used for some calculations for performance reasons. *ggplot2* 8. was used to create figures.

## Results and discussion

### The effect of PCR efficiency

**Fig. 1:**
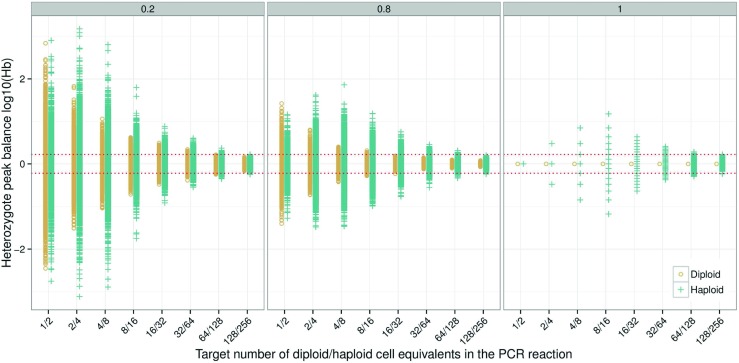
Simulation of 500 samples for diploid and haploid cells with PCR efficiencies of *p* *c* *r*_*e*_=0.20, *p* *c* *r*_*e*_=0.80 and *p* *c* *r*_*e*_=1.00, respectively. Stutters were not simulated. In this example, the heterozygote balance is affected only by the cell type and the PCR efficiency. This was achieved by setting all other parameters to one (*e* *x*_*e*_ = *p* *c* *r*_*a*_ = *c* *e*_*a*_=1.00). To obtain observations at low PCR efficiency for low-template, the detection threshold was set to zero (*c* *e*_*T*_=0), and no peak height scaling was applied (*c* *e*_*S*_=1), i.e. the number of molecules was used to calculate *H* *b*. *Circles* are diploid, *crosses* are haploid, and the *red dotted lines* denote the 0.6<*H* *b*<1.67 thresholds

Theoretical simulations to explore the effect of PCR efficiency on the heterozygote balance were performed (Fig. 1). Direct PCR was simulated at three efficiencies; *p* *c* *r*_*e*_=0.20, *p* *c* *r*_*e*_=0.80, and *p* *c* *r*_*e*_=1.00. Low PCR efficiency may be caused by inhibition (refer to the exhaustive review by [21] and [22] for details on mechanisms and solutions to overcome inhibition).

Simulations show increased heterozygote imbalance as the template DNA is reduced from optimal amounts (usually 0.5 to 1 ng). This has been shown in numerous publications, e.g. [23]. Conversely, increased template decreases the heterozygote imbalance until a minimum is reached. Adding more template beyond this point will not improve the balance further. Increased PCR efficiency also reduces the imbalance. Both alleles for diploid cells are perfectly balanced when the *p* *c* *r*_*e*_=1.00 (Fig. 1). However, this is not true for haploid cells [24].

If allelic copies are randomly drawn from a pool of haploid alleles that comprises equal number of (*a*, *b*) alleles at a heterozygous locus, this leads to a discrete distribution of possible ratios. For example, consider a DNA extract with four haploid genome copies with alleles *a* and *b*. There are only three possible copy number ratios that can be randomly drawn for a heterozygous (*ab*) locus: 1/3, i.e. one *a* and three *b*s, 2/2, and 3/1, with probabilities of 0.25, 0.375, and 0.25, respectively. A ratio 0/4 and 4/0, each with a probability of 0.0625, is also possible but will give rise to +- infinity when *l* *o* *g*_10_ is taken (for these combinations, alleles *a* and *b*, respectively, have dropped out, giving the appearance of a homozygote). This is further elaborated in the online supplement, Section B. For a mathematical model, simulations, and risk assessment of false homozygotes for diploid cells refer to [25]. The discrete or multi-modal nature of haploid peak height ratios is clearly visible at *p* *c* *r*_*e*_=1.00 with a small number of cells. As the PCR efficiency is reduced the multi-modality is smoothed as previously shown by [12]. As the number of haploid cells increases, the imbalance reaches a maximum at approximately 8–16 haploid cells. We call this the ‘diamond’ effect. The diamond effect is clearly visible at *p* *c* *r*_*e*_=1.00 and, to a lesser extent, at *p* *c* *r*_*e*_=0.80 (Fig. 1). The distribution for diploid cells has a funnel shape. This is also true for haploid cells at *p* *c* *r*_*e*_=0.20 (Fig. 1 left facet). With >16 cells and *p* *c* *r*_*e*_=1.00, the number of discrete possibilities becomes so tightly packed that the distribution can be considered continuous. Furthermore, the distribution for haploid cells converges towards the diploid distribution. A general threshold that is used by laboratories to denote a balanced locus is 0.6<*H* *b*<1.67 [26] (using Eq. 1).

For haploid cells, the probability of observing *H* *b* within the range accepted as ‘balanced’ (0.6<*H* *b*<1.67) and perfectly balanced (*H* *b*=1) is listed in Table 1. As the number of haploid cells increase, the probability of perfect balance reduces, while the probability of ‘accepted’ balance increases. [27] calculated the number of haploid cells needed to recover perfect balance with different values for the standard error.

**Table 1:** Theoretical probabilities of heterozygote balance within the accepted range (0.60≤*H* *b*≤1.67) and in perfect balance of different numbers of haploid cells as modelled by the Poisson distribution.

Haploid cells	*P*(accepted)	*P*(balanced)
2	0.500	0.500
4	0.375	0.375
8	0.711	0.273
16	0.790	0.196
32	0.890	0.140
64	0.967	0.099
128	0.997	0.070
256	1.000	0.050

The effect of low PCR efficiency is most noticeable for diploid cells, as the distribution of peak height ratios approaches the distribution for haploid cells. However, the distributions of heterozygote balance for diploid and haploid cells never converge completely. Not even when the PCR efficiency is reduced to *p* *c* *r*_*e*_=0.20 (Fig. 1).

### The effect of PCR aliquot

**Fig. 2:**
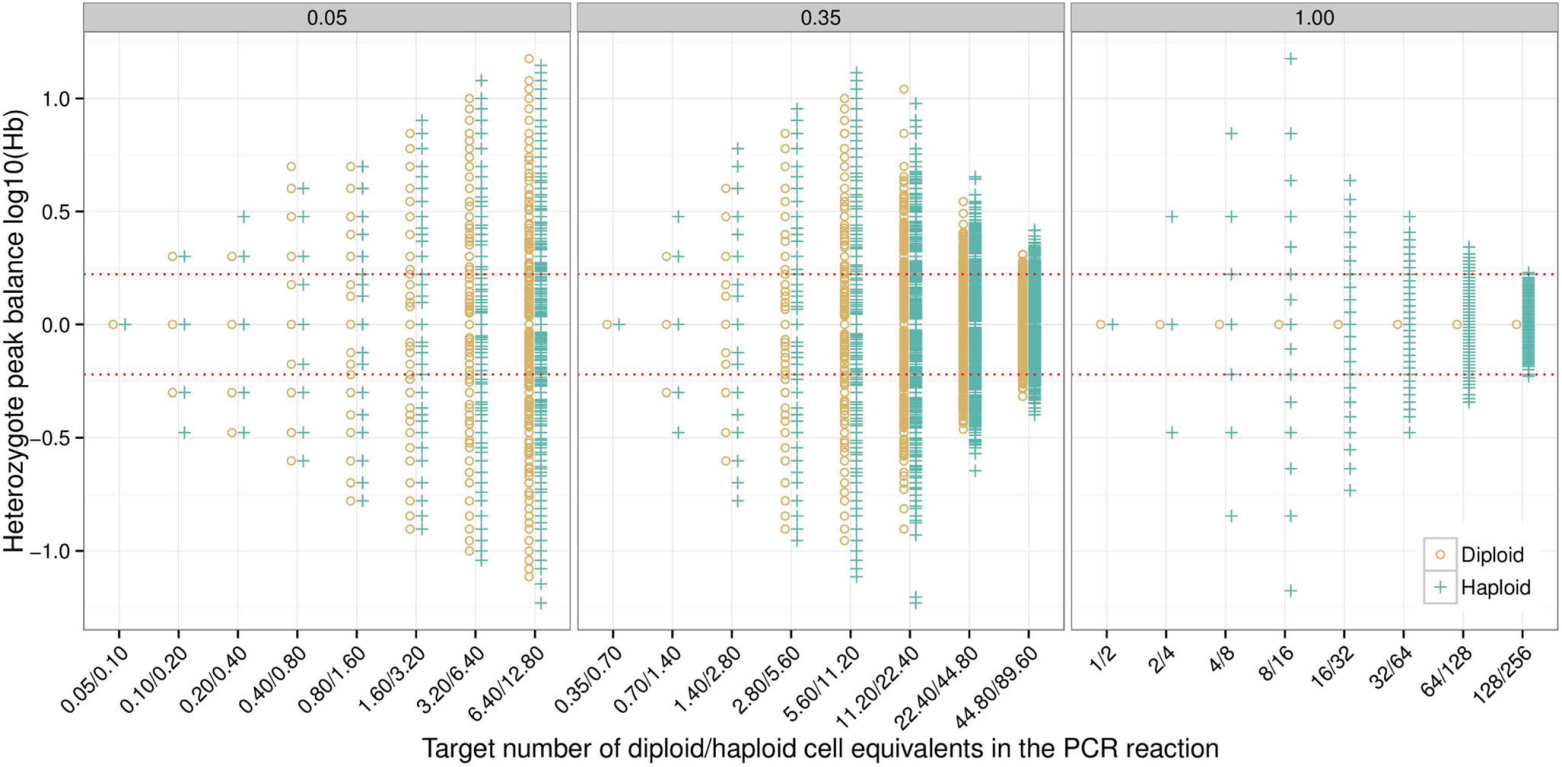
Simulation of 1500 samples for diploid and haploid cells with PCR aliquots of *p* *c* *r*_*a*_=0.05, *p* *c* *r*_*a*_=0.35 and *p* *c* *r*_*a*_=1.00, respectively. Stutters were not simulated. The heterozygote balance is affected only by the cell type and the PCR aliquot proportion. This was achieved by setting all other parameters to one (*e* *x*_*e*_ = *p* *c* *r*_*e*_ = *c* *e*_*a*_=1.00). To obtain observations at low PCR efficiency for low-template, the detection threshold was set to zero (*c* *e*_*T*_=0), and no peak height scaling was applied (*c* *e*_*S*_=1), i.e. the number of molecules was used to calculate *H* *b*. *Circles* are diploid, *crosses* are haploid, and the *red dotted lines*denote the 0.6<*H* *b*<1.67 thresholds

Pre-PCR random sampling of alleles is the main source of stochastic effects in low template samples [11, 18]. The aliquot forwarded to PCR is usually in the range 0.05 to 0.35 of the DNA extract^3^. Common reasons for a small aliquot is 1) the extraction method utilised requires a relatively large final volume, 2) to allow multiple PCR amplifications, or 3) a combination of these factors. To explore the effect of PCR aliquot proportions on heterozygote balance, theoretical simulations with fixed numbers of cells (1–128 diploid cells, and 2–256 haploid cells) in the DNA extract were performed (Fig. 2). Simulations of direct PCR *p* *c* *r*_*a*_=1.00, and aliquot proportions of *p* *c* *r*_*a*_=0.05 and *p* *c* *r*_*a*_=0.35 were compared. The difference in *H* *b* variance between *p* *c* *r*_*a*_=0.05 and *p* *c* *r*_*a*_=0.35 is large at the two highest DNA concentrations (64 and 128 diploid cells equivalent to 384 and 768 pg in the source DNA extract). However, the difference between distributions from diploid and haploid cells are small, and at *p* *c* *r*_*a*_=0.05, they are practically identical. Smaller aliquot proportions spread the possible outcomes of randomly selected alleles in diploid cells to the point where the distributions converge. The diamond effect is clearly visible for *p* *c* *r*_*a*_=0.35 (Fig. 2) where the average amount in the PCR tube approaches sub-cell levels. The variance is self-limiting because there is a limited number of possible copy number ratios for heterozygous loci at very small concentrations (as explained in ‘3The effect of PCR 3efficiency’).

### The effect of extraction efficiency

**Fig. 3:**
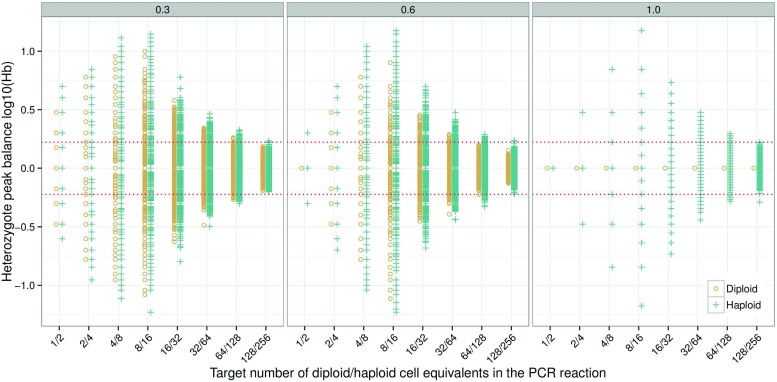
Simulation of 1000 samples for diploid and haploid cells with extraction efficiencies of *e* *x*_*e*_=0.30, *e* *x*_*e*_=0.60, and *e* *x*_*e*_=1.00, respectively. Stutters were not simulated. The heterozygote balance is affected only by the cell type and the extraction efficiency. This was achieved by setting all other parameters to one (*p* *c* *r*_*a*_ = *p* *c* *r*_*e*_ = *c* *e*_*a*_=1.00). To obtain observations at low PCR efficiency for low-template the detection threshold was set to zero (*c* *e*_*T*_=0), and no peak height scaling was applied (*c* *e*_*S*_=1), i.e. the number of molecules was used to calculate *H* *b*. *Circles* are diploid, *crosses* are haploid, and the r*ed dotted lines* denote the 0.6<*H* *b*<1.67 thresholds

The DNA extraction process contributes to the pre-PCR random sampling of alleles. Loss of DNA during the extraction process is unavoidable. The loss can be caused by transfer steps, incomplete cell lysis [28], incomplete cell elution [29], or other reasons mentioned in [30]. For one-tube extraction methods like Chelex [31], there is no loss of DNA due to transfer steps. For simplicity, in this paper we assumed that pure samples with small number of cells have an extraction efficiency of 100 %. Hence, the PCR aliquot proportion will be the only source of pre-PCR allele sampling. To explore the effect of extraction efficiency on *H* *b*, simulations were performed using *e* *x*_*e*_=0.30, *e* *x*_*e*_=0.60, and *e* *x*_*e*_=1.00 (Fig. 3). At *e* *x*_*e*_=1.00 (i.e. direct PCR) all alleles from diploid cells are in perfect balance, while alleles from haploid cells form discrete distributions. At *e* *x*_*e*_=0.30, the diploid and haploid *H* *b* distributions are roughly equal. However, at *e* *x*_*e*_=0.60, the difference between diploid and haploid cells are quite large implying that cell type has an effect on *H* *b* at high extraction efficiencies. As with changes in PCR efficiency (Fig. 1), it is observed that as the extraction efficiency decreases, the diamond shape widens at the lower end to become more funnel shaped (Fig. 3).

### Very low amounts of DNA lead to reduced heterozygote imbalance

Previous authors have determined that the variance for heterozygote imbalance increases as the amount of DNA decreases [18, 23, 32–35]. We have shown that this is only partially true. In fact, the reverse happens when the DNA concentration reaches a lower threshold. The theoretical reasoning and independent simulations to verify this is elaborated in the online supplement, Section B. The reason that it has not been previously noted is that the experimental design at very low levels of DNA is very difficult to accommodate. This is where simulation methods not only complement experiments, but can be used to inform experimental design by providing information about predicted behaviour.

### Serial dilutions vs. crime stains

For convenience, many laboratory experiments and validations are carried out using highly concentrated stock solutions of extracted DNA which is diluted in several steps to the desired target concentrations [18]. Then, the laboratory applies the measured characteristics (*H* *b*, stutter, etc.) to crime stains that are run routinely. However, dilution experiments do not strictly emulate the conditions in crime stains [36]. The purpose of the following simulations was to determine whether dilution experiments could be used instead of a much more complex experimental design that carries out assessments according to cell type while varying the number of cells per stain. We simulated serial dilutions according to [18] and compared them to simulated diploid and haploid crime stain samples (see ‘3Serial dilutions vs. 3crime stains’).

**Fig. 4:**
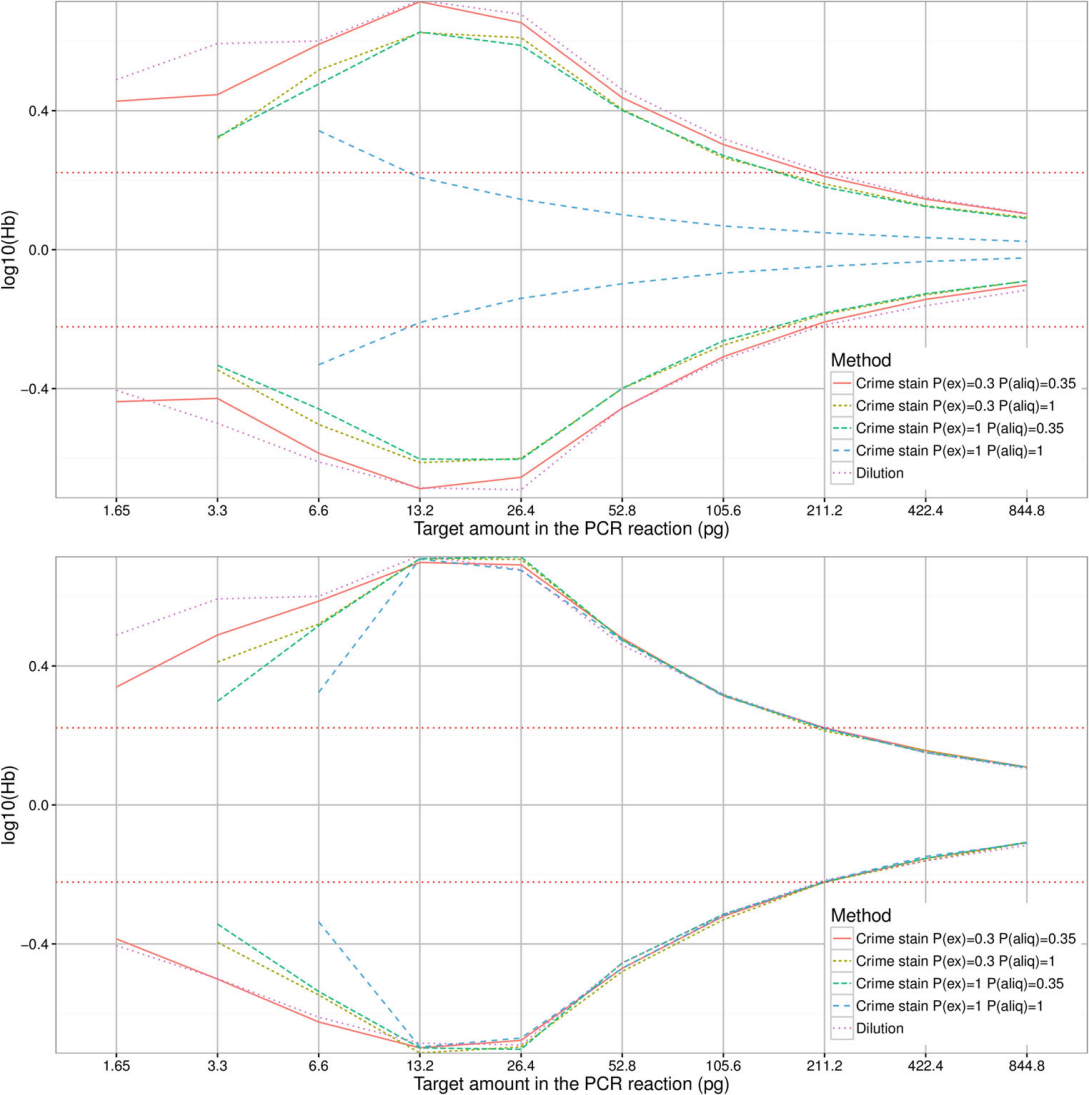
The 5th and 95th percentile *H* *b* of simulated serial dilution and simulated crime stains. Diploid crime stains (*top*) and haploid crime stains (*bottom*)

The 5th and 95th percentiles of *H* *b* are shown in Fig. 4. The simulated dilution reaches its maximum at two to four diploid cell equivalents of DNA (i.e. 13.2 to 26.4 pg). In comparison to simulated diploid crime stains (Fig. 4, top) the serial dilution appears to have roughly the same variance distribution, with the exception of direct PCR - a serial dilution from pristine and highly concentrated DNA does not accurately reflect direct PCR. Direct PCR has a very narrow funnel shaped distribution with a maximum at 1 diploid cell. The serial dilution more closely resembles the distributions from simulated haploid crime stains (Fig. 4, bottom). This has previously been pointed out in [27]. Down to approximately four haploid cell equivalents of DNA there is practically no difference between the simulated methods. The exception is a very low amount (<4) of haploid cells for direct PCR where the difference becomes larger with a decreasing number of haploid cells.

The difference between serial dilutions and crime stain samples, amplified using non-direct PCR methods to infer *H* *b* distributions is relatively small. This suggests that the use of serial dilutions is a reasonable approximation, which was also concluded in [34]. The exceptions are methods where both the extraction efficiency and the aliquot proportion are high, e.g. direct PCR, and the cell type is diploid. The ‘diamond’ effect is observed in the simulated data and suggests that the *H* *b* variance starts to decrease below two diploid, or four haploid, cell equivalents of DNA.

### Compromised crime stains

Degraded DNA is a common complication with forensic samples. Environmental factors such as humidity, bacteria, and ultraviolet light break down the DNA [37]. Longer DNA fragments are affected more than shorter DNA fragments causing increased imbalance [38] (see ‘3The effect of 3degradation’). The degradation can be modelled by an exponential curve [39]. Given two measurements of the DNA concentration in a single sample, using qPCR targets of different lengths, the probability of degradation per base pair *P*(*d* *e* *g*) can be calculated (Eq. 8). Inhibition of the Taq polymerase reduces PCR efficiency and increases the imbalance, the effect is greatest on high molecular weight fragments [40, 41]. Therefore the effect of inhibition is the same as degradation. Consequently, both inhibition and degradation can be modelled using the PCR efficiency and degradation parameters together. There are also other modes of inhibition e.g. DNA sequence specific inhibition, which are currently not modelled in *pcrsim*.

### The effect of degradation

DNA extracts from degraded tissue samples were quantified using two quantification kits with capability to measure degradation (i.e. the DNA concentration using two different target sizes). The result was used to estimate *P*(*d* *e* *g*), the probability that a single base pair is degraded, for each sample (derivation is explained later in this section). Table 2 shows the estimated probabilities, together with the DNA concentrations and the degradation index (*DI*) which is calculated according to Eq. 2:

2 $$ DI = \frac{C_{small}}{C_{large}}  $$

where *C*_*small*_ and *C*_*large*_ is the DNA quantity of the small and large target respectively. The degradation index is a useful indicator of size dependent quantities that are present in a crime sample. After calibration against the generated DNA profiles the *DI*, can be used to decide how to process the sample. The calculated *DI* differs between Quantifiler®; Trio and PowerQuant^TM^ because the target sizes are different. However the estimated *P*(*d* *e* *g*) parameter is reasonably similar, suggesting that it is kit independent. It is likely that only one calibration to EPGs is required for all quantification kits able to measure degradation if *P*(*d* *e* *g*) is used rather than *DI*. The degradation parameter can potentially be used as a standardized measure of degradation which would facilitate collaboration and inter-laboratory information exchange even if different kits are used.

**Table 2:** Concentrations (ng/*μ*l), degradation index (*DI*), and estimated degradation parameter (*P*(*d* *e* *g*)) for nine degraded tissue samples based on quantification by Quantifiler®; Trio DNA Quantification Kit (QT) and PowerQuant^TM^ System (PQ). Target amplicon size in base pairs is 80 and 84 for the small targets, and 214 and 294 for the large targets. A degradation index *DI* is calculated by dividing the small autosomal target DNA concentration by the large autosomal target DNA concentration (Eq. 2)

Sample	QT:80	PQ:84	QT:214	PQ:294	QT:DI	PQ:DI	QT:P(deg)	PQ:P(deg)
D1	119.710	127.850	83.6300	27.7100	1.4	4.6	0.0027	0.0073
D2	0.058	0.003	0.0009	NA	64.9	NA	0.0307	NA
D3	45.605	59.950	1.5170	1.8600	30.1	32.2	0.0251	0.0164
D4	0.437	0.547	0.1357	0.0540	3.2	10.1	0.0087	0.0110
D5	0.256	0.333	0.0111	0.0042	23.1	79.2	0.0231	0.0206
D6	0.098	0.119	0.0079	0.0024	12.4	49.5	0.0186	0.0184
D7	0.284	0.292	0.0036	0.0034	78.8	86.0	0.0321	0.0210
D8	1.437	1.712	0.0900	0.0859	16.0	19.9	0.0205	0.0141
D9	31.510	55.710	0.0565	0.0077	557.7	7235.1	0.0461	0.0414

**Fig. 5:**
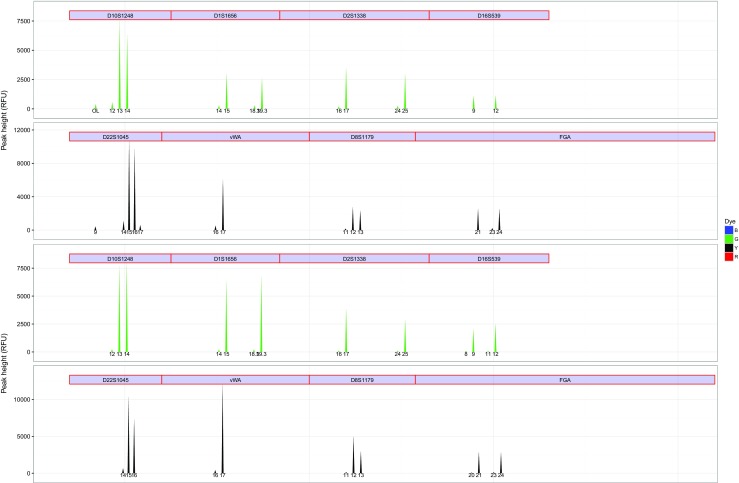
Comparison of an observed (*top*) and simulated (*bottom*) degraded ESX 17 Fast profile. Only the green and yellow dye is shown for visibility. A degradation parameter of *P*(*d* *e* *g*)=0.0023 was used

The estimated *P*(*d* *e* *g*) was plugged into the PCR simulator. Figure 5 shows observed and simulated EPG’s of a degraded sample. The overall characteristics of the degraded DNA profile are very similar.

Degradation is a consequence of random DNA cleavage (refer to the exhaustive review by [37] for details on mechanisms and consequences of degradation). Consider a fragment of DNA that is *x* bases long. It makes no difference whether 1 or more cleavages occur within the fragment of interest, as the fragment will fail to amplify no matter where the DNA was cleaved. As a result the fragment will not be visualized.

Usually, there are multiple copies of DNA. Degradation can be related to drop-out. Allele drop-out occurs either because no copies are amplified (i.e. no molecules present in the PCR reaction), or because the fluorescence signal fails to reach the threshold value of the CCD detector of the capillary electrophoresis machine (i.e. insufficient number of molecules present in the PCR reaction).

We can generalize that the chance of drop-out is related to the number of template DNA copies - the fewer the number of copies the lower the fluorescence after PCR. Thus probability of drop-out of a single copy of DNA can be characterized by Eq. 3, assuming independence between the base pairs of the copy, to estimate the chance of cleavage of a molecule of *x* bases:

3 $$ P(drop_{1})=1-(1-P(deg))^{x}  $$

where *P*(*d* *r* *o* *p*_1_) is probability of drop-out. We can write the probability that a fragment of size *x* is intact (i.e. not degraded) and available for amplification (Eq. 4):

4 $$ P(!drop_{1})=(1-P(deg))^{x}  $$

However, it is necessary to evaluate the probability of cleavage of DNA as a function of *n* copies of DNA. This is defined by the binomial probability in Eq. 5:

5 $$ P(drop_{n})=1-(1-P(drop_{1}))^{n}  $$

We make the same assumptions as [42] and use a constant probability of cleavage across the fragment sequence. Similar to [42], we assume a log-linear relationship between concentration *c*(*x*), fragment length *x*, and the probability of no degradation, i.e. Eq. 6:

6 $$ ln(c(x))=ln(H)+ln(1-P(deg))x  $$

**Fig. 6:**
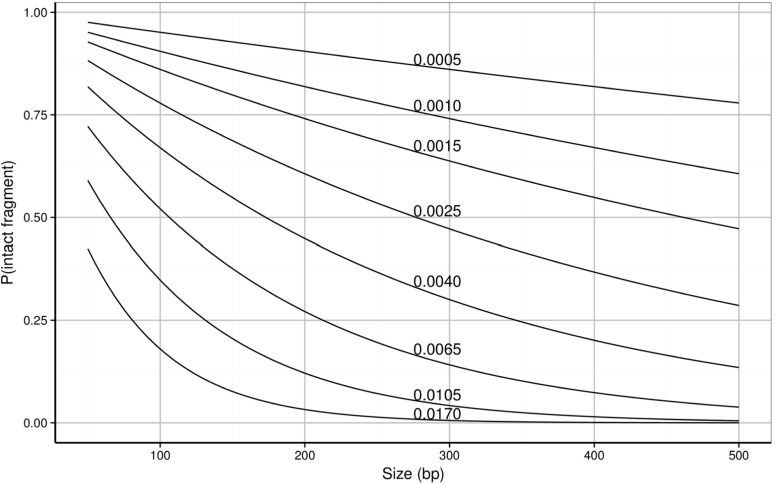
Probability of intact fragment available for amplification as a function of fragment length for different values of the degradation parameter (0.0005–0.0170)

From this follows Eqs. 7 and 8:

7 $$ ln(1-P(deg))=\frac{ln(c(x_{2}))-ln(c(x_{1}))}{x_{2}-x_{1}}  $$

8 $$ P(deg)=1-e^{\frac{ln(c(x_{1}))/ln(c(x_{2}))}{x_{1}-x_{2}}}  $$

where *x*_1_<*x*_2_ and *c*(*x*_2_)≤*c*(*x*_1_). We can formalize this as the probability of 5$^{\prime }$ cleavage of the affected base. Therefore, given *P*(*d* *e* *g*) the number of intact fragments of any fragment length can be estimated. Figure 6 shows the probability of intact fragments as a function of fragment length, for different values of the degradation parameter using Eq. 4.

Modern human real-time DNA quantification kits (e.g. Quantifiler®; Trio DNA Quantification Kit and PowerQuant^TM^ System) often come with the ability to measure the degree of degradation for each sample. This is accomplished by adding a second longer target to measure the total human DNA. Usually a 200–300 base pair fragment (*x*_2_) is generated from the longer target, while the shorter generates a 70–150 base pair fragment (*x*_1_).

**Fig. 7:**
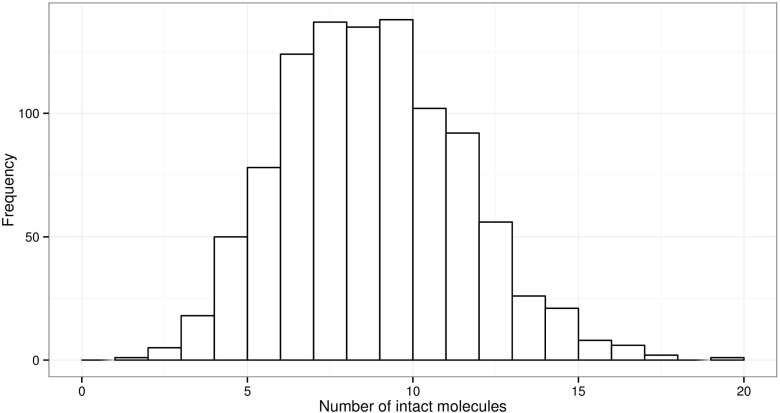
1000 simulations *N*=167 copies of a 300 bp fragment with *P*(!*d* *r* *o* *p*)=0.05 probability of being intact, corresponding to approximately *P*(*d* *e* *g*)=0.0099. The range of intact fragments is 1–19, which may fail to produce sufficient PCR product to trigger a signal

To simulate degradation, the probabilities that each allele (i.e. fragment length) is complete, and thereby available for amplification, is calculated. Then a binomial selection of molecules with the previously calculated probabilities is applied ‘post PCR’ (Eq. 9):

9 $$ N_{intact} = Bin(N_{molecules}, P(!drop))  $$

where *N*_*molecules*_ is the number of molecules of each allele after PCR amplification, and *N*_*intact*_ is the number of intact molecules. To illustrate, we simulate degradation of a fragment of 300 bases, 1 ng total DNA, corresponding to 167 haploid copies (Fig. 7) using the binomial distribution *B* *i* *n*(*N*=167,*P*=0.05).

This results in a population of intact fragments that can be amplified, but with this particular example, where *P*(!*d* *r* *o* *p*)=0.05, there are between 1 and 19 undegraded copies derived from 1 ng. A DNA fragment will only be visualized if there are sufficient molecules present to trigger the capillary electrophoresis machine’s CCD camera. For 28 cycles, approximately 30 haploid copies (ca 90 pg) are required before sufficient PCR product is available to trigger a signal [43], whereas for 34 cycles, just one molecule (ca 3 pg in a haploid cell) is needed to produce sufficient signal [44].

Therefore optimization of systems when degraded DNA is analysed, cannot be considered without a concurrent consideration of the effect of PCR cycle number.

**Fig. 8:**
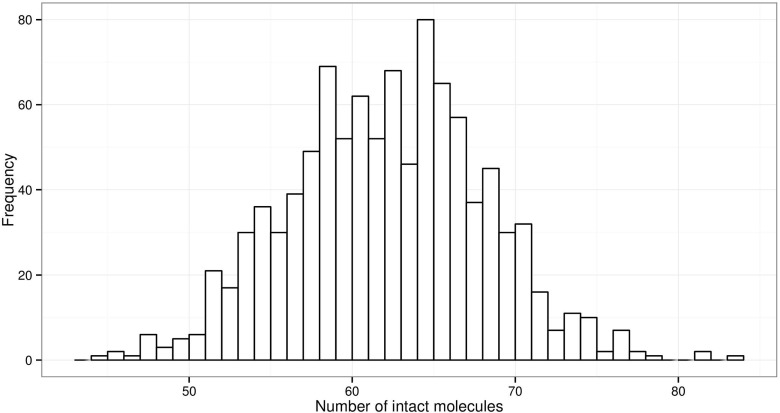
1000 simulations *N*=167 copies of a 100 bp fragment with *P*(!*d* *r* *o* *p*)=0.37 probability of being intact, corresponding approximately to *P*(*d* *e* *g*)=0.0099. The range of intact fragments is 44–83, which normally result in sufficient PCR product to trigger a signal

We repeated the simulation with a smaller fragment size of 100 bases (Fig. 8). Using the same degradation parameters, a fragment that is just 100 bases has a chance of 37 % of surviving, thus a nanogram of DNA from diploid cells will have between 44 and 83 intact molecules of each allele (approximately 264 to 498 pg). The threshold is always exceeded even at 28 cycles.

#### Heterozygote balance and the ‘diamond effect’

**Fig. 9:**
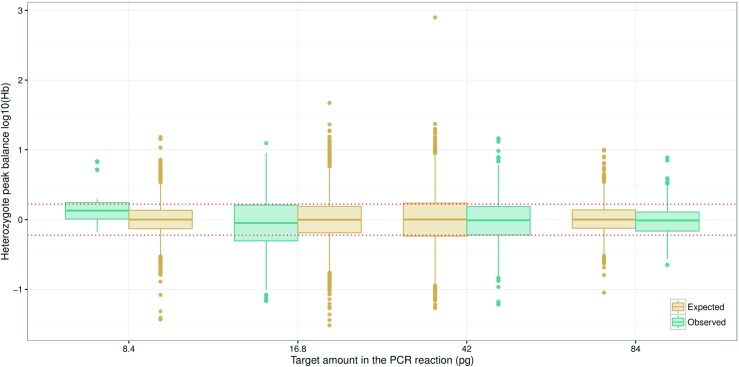
Heterozygote balance from a total of 200 simulated ESX16 samples compared to observed data from another laboratory. The average amount was estimated to 8.4, 16.8, 42, and 84 pg, respectively (Table 4 in online supplement, Section A). The simulation was performed with average amounts of approximately 2.4, 19.5, 31.7, and 63.3 pg of DNA in the PCR reaction. Those amounts gave the best fit to observed data using the approach described in the online supplement, Section A

Real data were analysed to verify the ‘diamond effect’ predicted by simulations. Previous experiments showed that heterozygote balance was mainly affected by the amount of DNA rather than the number of PCR cycles or the CE injection time [16]. This has also been confirmed in for example [45]. Based on this knowledge, observations from different injection times and different number of PCR cycles were combined for each target amount of DNA. This increased the number of observations for the lowest amount to allow meaningful comparisons. Heterozygote balance from simulated (expected) compared with real (observed) data is shown in Fig. 9. The data visually support the contention that the variance decreases for very low amounts of DNA, as predicted. The simulation was performed using average amounts of approximately 2.4, 19.5, 31.7, and 63.3 pg of DNA in the PCR reaction. A range of different amounts were tried in order to find the input amounts giving the best fit to observed data (further described in online supplement, Section A). The uncertainty in experimental quantification results are large since they are also subject to stochastic effects and this is probably the main reason that adjusted input amounts were required to provide good fit. The adjusted amounts predicted by the ‘fitted’ models are all within the range of the quantification results and are near the median values (Table 4 in online supplement, Section A). It is possible that *H* *b* may be a useful aid to determine quantity of DNA. However, this is not pursued further here as more work is required to verify a useful method. Other parameters which will influence the simulations are the assumed PCR efficiency, the CE detection threshold and peak height scaling. Calibration of *pcrsim* using the same CE instrument that produced the data would likely improve the accuracy as there is variation between instruments.

### Implications for casework

#### Maximizing the chance to obtain a complete profile

[24] calculated that at least 15 haploid cells are required for a complete DNA profile (≥99.9 *%* confidence) at 15 loci and indeed [46] confirmed that it is possible to produce complete Identifiler®; profiles from only 15 sperm cells using Laser Capture Microdissection (LCM). For robust LCM approximately 30 sperm cells are needed [47] (refer to Vandewoestyne et. al. for a review of LCM techniques [48]). For a specific DNA extract, the aliquot proportion determine the amount of DNA forwarded to PCR and, hence, the chance to obtain a complete DNA profile. Table 3 shows the theoretical numbers of cells required in DNA extracts, before the aliquot is taken, to result in a given amount of DNA in the PCR reaction. Theoretically, provided enough diploid cells are selected to pass the fluorescence detection threshold, this will always result in a complete DNA profile for direct PCR (i.e. *p* *c* *r*_*a*_=1.00). When an aliquot of an extract is used, a random selection of alleles takes place (because of dissociated chromosomes), resulting in reduced chance to obtain a complete profile. In essence, this is similar to samples of haploid cells.

**Table 3:** Theoretical number of cells (rounded up) required in the DNA extract, before an aliquot (of 5, 35, and 100 %) is taken, to obtain a certain average amount (pg) in the PCR reaction.

5 %	35 %	100 %	Amount
20	3	1	6
40	6	2	12
80	12	4	24
160	23	8	48
320	46	16	96
640	92	32	192
1280	183	64	384
2560	366	128	768

#### Inhibiting substances increase heterozygote imbalance

[49] used LCM to collect 15 to 150 FISH labelled diploid cells for direct PCR, using 28 cycles and the Identifiler®; PCR Amplification Kit, and investigated the heterozygote balance. Although one-tube extraction and direct PCR (i.e. *e* *x*_*e*_ = *p* *c* *r*_*a*_=1.00) should minimise stochastic effects, it was concluded that there was no improvement^9^ in peak height balance compared to single-source crime scene samples analysed in a study conducted by [50]. Further comparison with ‘Christmas Tree’ stained cells indicated that the FISH process has a negative impact on peak balance.

#### Haploid versus diploid cells

**Fig. 10:**
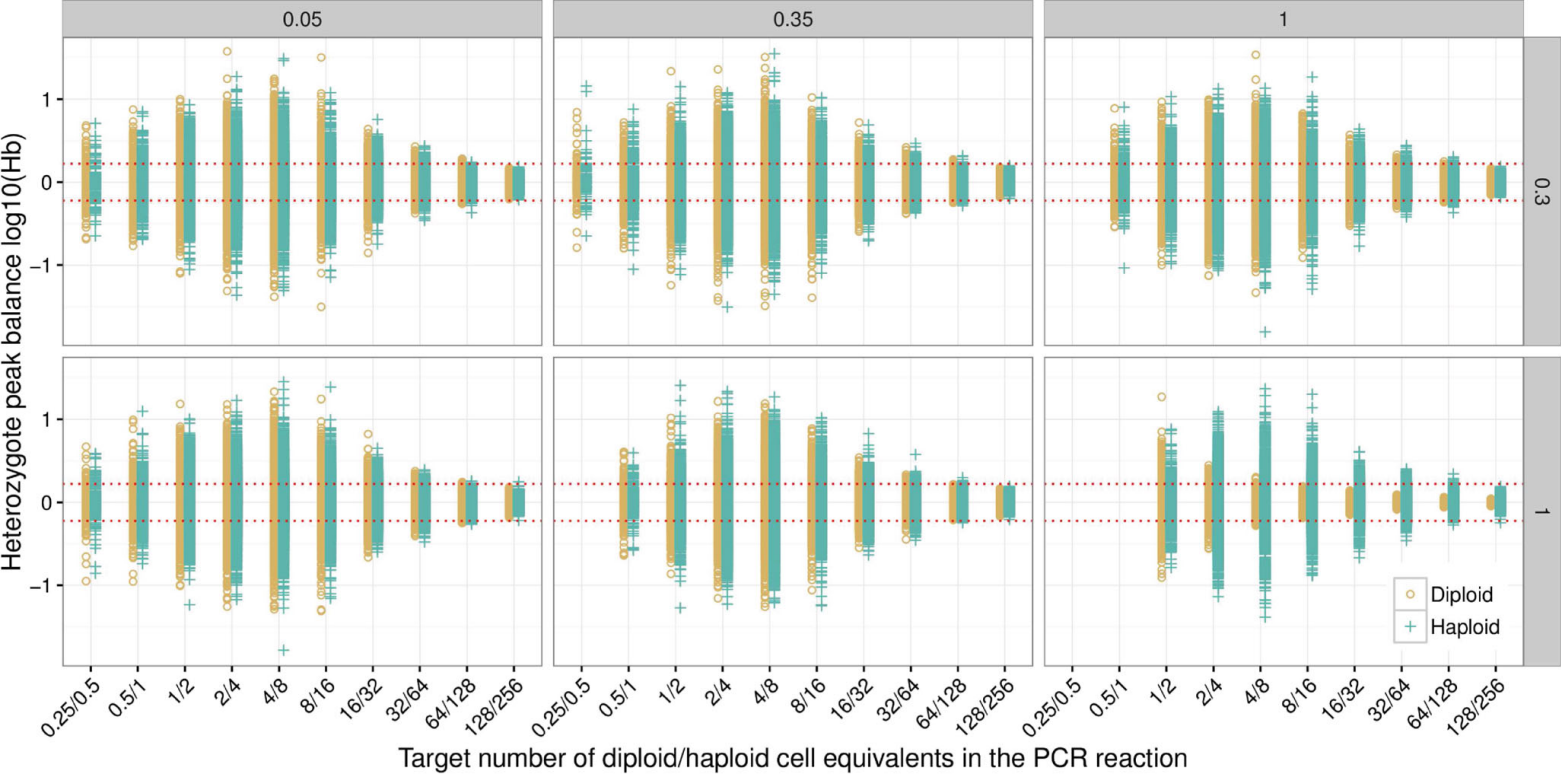
Heterozygote balance from 250 simulated samples with *e* *x*_*e*_=0.30 and *e* *x*_*e*_=1.00, and *p* *c* *r*_*a*_=0.05, *p* *c* *r*_*a*_=0.35, and *p* *c* *r*_*a*_=1.00 in combination. PCR parameters were *p* *c* *r*_*c*_=30 and *p* *c* *r*_*e*_=0.90. Stutters were not simulated. Capillary electrophoresis parameters were *c* *e*_*a*_=1.00, *c* *e*_*T*_=0, *c* *e*_*S*_=1, i.e. the number of molecules was used to calculate *H* *b*. The *red dotted lines* denotes the 0.6<*H* *b*<1.67 thresholds

Concurrently varying extraction efficiency and PCR aliquot proportion (Fig. 10) shows that for normal casework (with extraction efficiencies of up to at least 30 % and aliquot proportions up to 35 %) the distributions of heterozygote balance for diploid and haploid cells are practically identical. Haploid cells then represent the worst case scenario for diploid cells (consider Figs. 1, 2, and 3). However if the extraction efficiency and the aliquot proportion are both relatively large, i.e. direct PCR, differences in haploid/diploid heterozygote balance distributions should be considered in models that evaluate complex DNA evidence.

## Conclusions

It has long been established that the variance in heterozygote balance increases as the amount of DNA is reduced. Using simulations, we have shown that the distribution is in fact diamond shaped. As the amount of DNA decreases, the variance increases until a maximum is reached. The variance starts to decrease at very low amounts of DNA (50 pg or less, depending on PCR efficiency, aliquot proportion, and extraction efficiency) and the distributions become multi-modal rather than continuous. This was also confirmed by experimental data. In theory, under optimal conditions, the alleles in amplified diploid cells will be in perfect balance. However the extraction process, aliquot proportion, and amplification efficiency introduces variance. Direct PCR is preferred for optimal allele balance and sensitivity and has been successfully implemented for certain casework samples [51, 52]. Simulations show that for direct PCR, haploid and diploid cells have different heterozygote balance distributions. This may need to be accounted for in some statistical models that are used to evaluate DNA evidence. However, direct PCR is not widely implemented (and may not always be suitable). With realistic extraction efficiencies and aliquot proportions the difference between *H* *b* variances is negligible. Consequently, diploid cells can be used in validation to determine characteristics of *H* *b* also for haploid cells. Simulations also suggest that diluted DNA extracts, which are commonly used in validations exercises are an acceptable approximation to crime stain samples (provided that care is taken to use large volumes) except for direct PCR methods or very low levels of DNA. Our results suggest that simulations of crime stains are preferred over dilutions when the average amount of DNA in the PCR reaction approaches sub-cellular amounts.

We have exemplified that the number of PCR cycles is a key factor to consider when degraded DNA is analysed. If the probability of degradation per base pair is used as a metric, rather than degradation indexes, the measure becomes kit independent. With knowledge of the degradation parameter the resulting characteristics of the DNA profiles can be predicted by simulation.

## Electronic supplementary material

Below is the link to the electronic supplementary material.

(PDF 4.48 MB)

(ZIP 8.17 MB)

## Footnotes

1 “Evaluation of DNA Extraction Efficiency” presented by Erica L.R. Butts, 65th American Academy of Forensic Sciences (February 2013)

2
http://cran.r-project.org/web/packages/pcrsim/index.html, accessed 08.04.2016

3 Personal communication with 5 European laboratories.

4 For details see ‘Calibrate pcrsim 1.0.0’ at https://sites.google.com/site/forensicapps/pcrsim.

5
http://www.r-project.org/, accessed 08.04.2016

6
https://github.com/Rdatatable/data.table/wiki, accessed 08.04.2016

7
http://plyr.had.co.nz/,accessed08.04.2016

8
http://ggplot2.org/,accessed 08.04.2016

9 The metric used was the average peak height where the central 0.95 quantile crosses the 60 % threshold.
