# Supplementary material for: Characterization of degradation and heterozygote balance by simulation of the forensic DNA analysis process
Source: Int J Legal Med. 2016 Nov 3;131(2):303–17. doi: 10.1007/s00414-016-1453-x (PMC5306348; doi:10.1007/s00414-016-1453-x)
Supplement: Supplementary file 1 — (PDF 4.48 MB) [file 414_2016_1453_MOESM1_ESM.pdf]

Electronic supplement material

Characterization of degradation and heterozygote balance by simulation of the forensic DNA analysis process (Oskar Hansson, Thore Egeland, Peter Gill)

A Input values for the simulation in section *Heterozygote balance and the 'diamond effect'*

Table 1 shows the human DNA quantification result for the experimental data used in Hedell et al. [16]. Section *Heterozygote balance and the 'diamond effect'* attempts to replicate the observed heterozygote balance distribution by simulation. Simulation using the estimated amounts, provided in Table 4, did not fit well with the observed data. Possible reasons are discussed in section *Heterozygote balance and the 'diamond effect'*. The process of finding the best fit to use in the comparison (Figure 9) is outlined below:

1. Simulation was performed over a range of 0.125 to 16 diploid cells using conditions that mimic the process generating the data. The range was created using the R command: `2^seq(-3, 4, by=0.1)`, which produce smaller changes for low amounts.
2. The log10 heterozygote balance was calculated for the observed data and simulated samples.
3. Homozygotes, heterozygotes separated by only one repeat, Nan values, and markers with mean peak height > 10000 RFU were removed from the dataset.
4. The standard deviation of heterozygote balance and the number of observations were calculated for each amount.
5. The squared difference between the simulated and observed standard deviation of the heterozygote balance was calculated for each estimated amount.
6. For each estimated amount an ordered list with increasing squared difference was produced (Figure 1).

7. Simulated amounts of the same order of magnitude as the estimated amounts, and with the least squared difference were selected (marked with yellow in Figure 1) for the comparison. The results are presented in Figure 9.

Table 1 Quantification results. Three replicates (rep.1 - rep.3) from each target dilution (8 to 84 pg) was quantified (Sample). Human DNA concentration in ng/µl (Concentration), median (Median) and average concentration (Average) are given. Two replicates did not have measurable DNA concentration (NA). The lower end of the standard curve was 0.006 ng/µl and values below this have been extrapolated. Therefore Hedell et al. [16] estimated the concentrations of the three lower concentration based on the sample with the highest concentration (Estimate). Finally the minimum and maximum amount in pg of DNA in the PCR reaction based on the range of concentrations have been calculated (Range).

Sample	Concentration	Median	Average	Estimate	Range
84.rep.1	0.0134	0.0070	0.0084	84	48-134
84.rep.2	0.0070				
84.rep.3	0.0048				
42.rep.1	0.0065	0.0033	0.0043	42	32-65
42.rep.2	0.0033				
42.rep.3	0.0032				
17.rep.1	0.0052	0.0016	0.0028	16.7	15-52
17.rep.2	0.0015				
17.rep.3	0.0016				
8.rep.1	NA	NA	0.0010	8.4	NA-32
8.rep.2	NA				
8.rep.3	0.0032				

rank	experiment	amount	sd	n	(est-sim)^2
1	Estimated	84.00	0.2168	1338	0.00E+00
2	Simulated	63.34	0.2148	3793	3.75E-06
3	Simulated	1.22	0.2187	339	3.87E-06
4	Simulated	1.50	0.2132	529	1.24E-05
5	Simulated	59.09	0.2212	3978	2.01E-05
6	Simulated	1.06	0.2101	214	4.40E-05
7	Simulated	0.75	0.2267	91	9.81E-05
8	Simulated	1.31	0.2271	336	1.07E-04
9	Simulated	1.61	0.2284	464	1.36E-04
10	Simulated	67.88	0.2036	3603	1.74E-04

rank	experiment	amount	sd	n	(est-sim)^2
1	Estimated	42.00	0.3233	1219	0.00E+00
2	Simulated	7.39	0.3257	4303	5.31E-06
3	Simulated	6.43	0.3206	3804	7.34E-06
4	Simulated	31.67	0.3159	5643	5.58E-05
5	Simulated	29.55	0.3338	5763	1.09E-04
6	Simulated	7.92	0.3350	4706	1.35E-04
7	Simulated	6.89	0.3108	3993	1.57E-04
8	Simulated	8.49	0.3372	4936	1.93E-04
9	Simulated	27.57	0.3379	5939	2.13E-04
10	Simulated	6.00	0.3081	3761	2.34E-04

rank	experiment	amount	sd	n	(est-sim)^2
1	Estimated	16.80	0.3777	712	0.00E+00
2	Simulated	19.49	0.3747	6426	9.13E-06
3	Simulated	16.97	0.3711	6403	4.33E-05
4	Simulated	18.19	0.3697	6417	6.39E-05
5	Simulated	13.78	0.3684	6232	8.67E-05
6	Simulated	15.83	0.3657	6382	1.45E-04
7	Simulated	12.00	0.3636	6007	2.00E-04
8	Simulated	20.89	0.3631	6359	2.13E-04
9	Simulated	14.77	0.3619	6359	2.50E-04
10	Simulated	22.39	0.3567	6277	4.40E-04

rank	experiment	amount	sd	n	(est-sim)^2
1	Estimated	8.40	0.2477	44	0.00E+00
2	Simulated	2.44	0.2441	1063	1.33E-05
3	Simulated	3.22	0.2516	1511	1.52E-05
4	Simulated	1.40	0.2429	331	2.32E-05
5	Simulated	48.00	0.2527	4584	2.42E-05
6	Simulated	1.98	0.2424	659	2.84E-05
7	Simulated	2.27	0.2412	848	4.30E-05
8	Simulated	1.72	0.2408	508	4.81E-05
9	Simulated	51.45	0.2404	4354	5.41E-05
10	Simulated	2.12	0.2394	851	6.96E-05

Fig. 1 The top ten of the ranked lists produced for each estimated (est) amount of DNA where the estimated amount was derived from the original quantification (Table 4) and the simulated (sim) result was the best fit of the model (section *Heterozygote balance and the 'diamond effect'*). Since the distribution of *Hb* is diamond shaped, high and low amounts will show similar *Hb* distributions and are therefore mixed in the ranked list. The fact that simulations with similar amounts are not ordered by amount is likely caused by stochastic effects. Two criteria were used to identify the 'best fit': 1) the squared difference (est-sim)ˆ2 of the *Hb* variance should be minimized, and 2) the simulated amount should be of the same order of magnitude as the estimated amount. The simulated amounts 63.3, 31.7, 19.5, and 2.4 were used instead of the estimated amounts 84, 42, 16.7, and 8.4 as they provided a better fit to the observed data. The result is presented in Figure 9.

B Random sampling of alleles

The 'diamond effect' can be theoretically derived from the following reasoning. If allelic copies are randomly drawn from a pool of haploid alleles that comprises equal number of (a, b) alleles at a heterozygous locus, this leads to a discrete distribution of possible ratios. For example, if there are two haploid genome copies with alleles a and b in the DNA extract, there are only one possible copy number ratio that can be randomly drawn for a heterozygous (ab) locus: 1/1 with a probability of 0.5. A ratio 0/2 and 2/0, each with a probability of 0.25, is also possible but will give rise to a Hb of 0 and infinity. For these combinations, alleles a and b respectively have dropped out, giving the appearance of a homozygote with a total probability of 0.5. If there are three haploid genome copies there are only two possible copy number ratios: 1/2, and 2/1, each with a probability of 0.375. The probability of 0/3 and 3/0 is 0.125 each. Hence, the total probability of obtaining a false homozygote is reduced from 0.5 to 0.25. The scenario with four haploid genome copies are described in section *The effect of PCR efficiency*. The possible outcomes for a and b alleles when sampling up to six molecules are shown in Table 2 with the corresponding probabilities shown in Table 3. Figure 2 show the observed ratio between alleles from simulations ordered by total number of drawn molecules. The observed ratio start from 1 for the lowest possible number of sampled molecules required to observe both alleles for a heterozygote, namely 2. Increasing the number of sampled molecules unavoidable leads to an increase in possible copy number ratios. However, the probability of obtaining the extreme ratios decrease (shown by the solid lines and weak points). Thus, the range of observed Hb reaches a tipping point at 7 sampled molecules, where the observed range start to decrease. Extreme values are still possible but with a lower probability. This is the 'diamond effect' demonstrated here by a simple binomial simulation. It support the results generated by *pcrsim*.

Why is the 'diamond effect' so difficult to observe experimentally? For low-template DNA extracts it is true that, with a small aliquot you will more often end up with nothing in the PCR reaction, while if the aliquot is large both allele a and b will more often be

sampled. This is shown by binomial simulation in Figure 3. It is indeed difficult to sample both alleles at low concentrations. This is why the 'diamond effect' is difficult to observe.

Table 2 The possible outcomes in number of a and b alleles when sampling different number of molecules (1-6) and the corresponding theoretical ratio (Hb). This is a numerical representation of the leftmost part of the graphs in Figure 2.

Hb	1	2	3	4	5	6
Inf	1/0	2/0	3/0	4/0	5/0	6/0
5.00						5/1
4.00					4/1	
3.00				3/1		
2.00			2/1			4/2
1.50					3/2	
1.00		1/1		2/2		3/3
0.67					2/3	
0.50			1/2			2/4
0.33				1/3		
0.25					1/4	
0.20						1/5
0.00	0/1	0/2	0/3	0/4	0/5	0/6

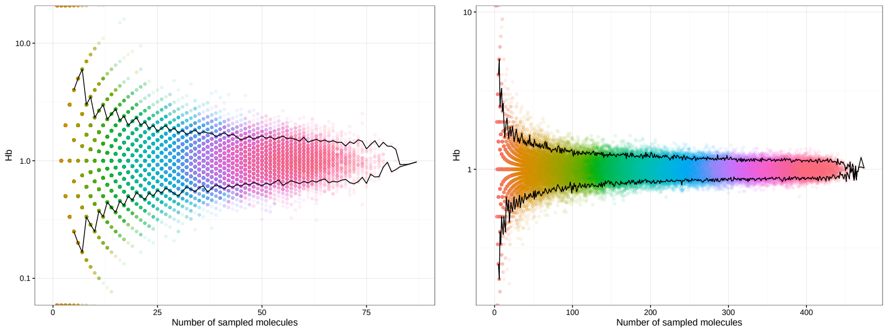

Fig. 2 For DNA extracts containing 10-600 molecules (in steps of 10) each of allele a and b respectively, an aliquot of 0.05 (left) and 0.35 (right) was taken from each DNA extract. This was repeated 1000 times. The ratio a/b (Hb) was plotted by the total number of sampled molecules. Points were plotted with 90% transparency such that 10 overlapping points is needed for completely opaque colour. The colour gradient is derived from the DNA concentration in the extracts. The 5th and 95th percentile is indicated by the solid line.

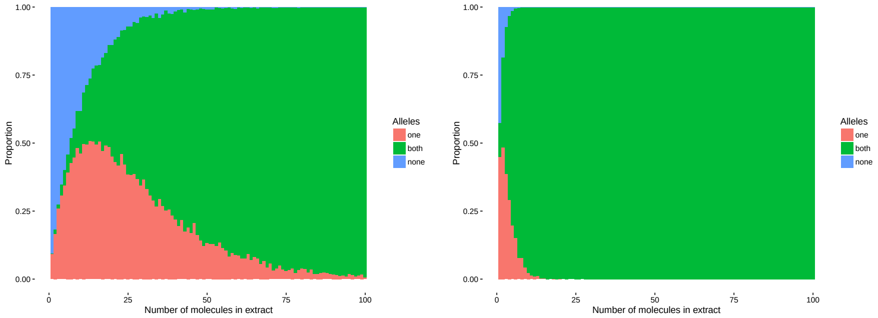

Fig. 3 For DNA extracts containing 1-100 molecules (in steps of 1) each of allele a and b respectively, an aliquot of 0.05 (left) and 0.35 (right) was taken from each DNA extract. This was repeated 1000 times. The proportion when none, one, or both alleles were obtained was plotted by the number of molecules in the DNA extract. This illustrates that a small aliquot often result in none or just one sampled allele type in the PCR reaction for low-template samples, while this is seldom the case for a large aliquot.

Table 3 The expected probability of the possible outcomes, shown in Table 3, when sampling different number of molecules (1-6) and the corresponding ratio (Hb). This is a numerical representation of the leftmost part of the graphs in Figure 2.

Hb	1	2	3	4	5	6
Inf	0.5	0.25	0.125	0.0625	0.03125	0.015625
5.00						0.09375
4.00					0.15625	
3.00				0.25		
2.00			0.375			0.234375
1.50					0.3125	
1.00		0.5		0.375		0.3125
0.67					0.3125	
0.50			0.375			0.234375
0.33				0.25		
0.25					0.15625	
0.20						0.09375
0.00	0.5	0.25	0.125	0.0625	0.03125	0.015625

C Degraded samples

The degraded sample in Figure 5 was simulated using *pcrsim* (version 1.0.0) with the following parameters.

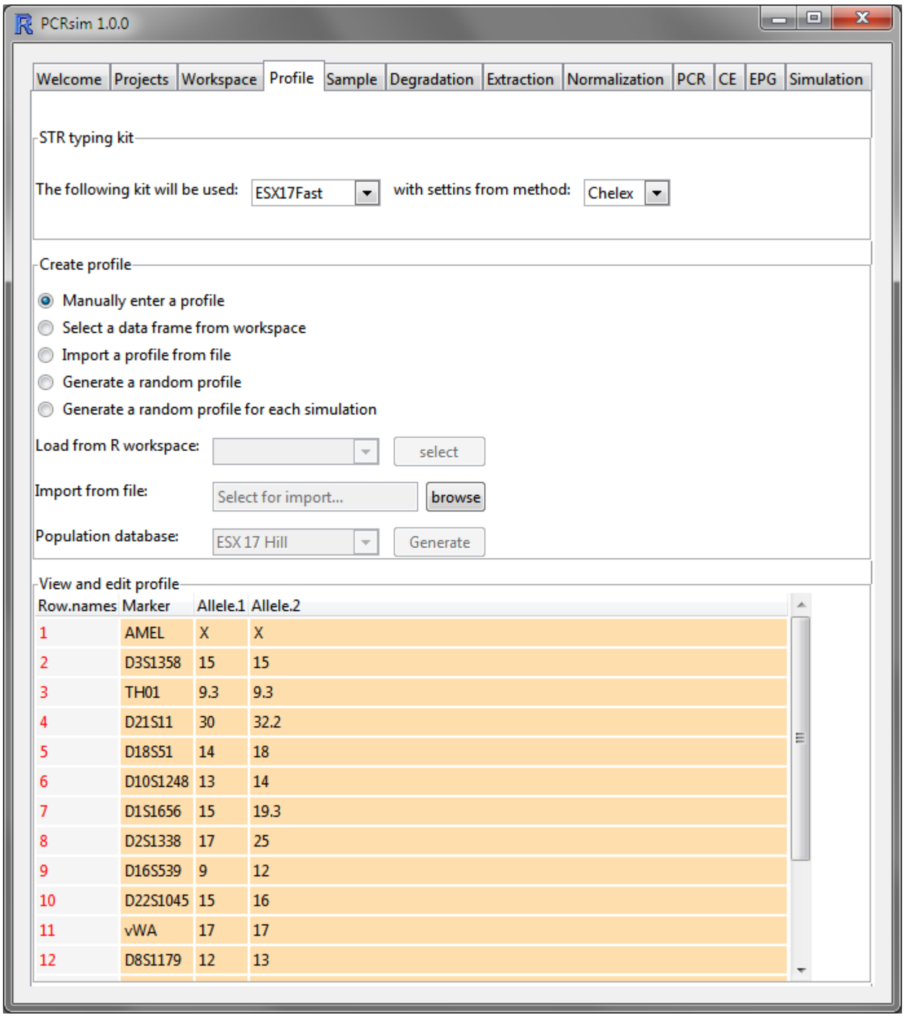

Fig. 4 The Profile tab.

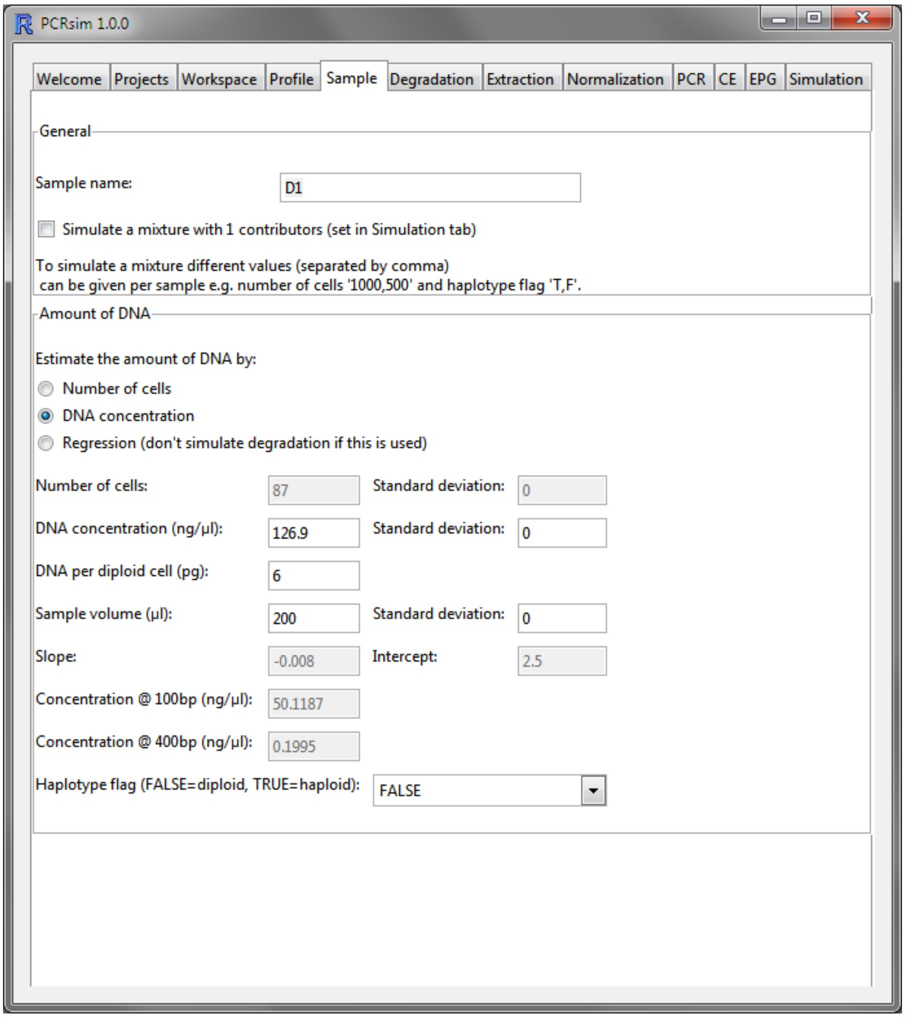

Fig. 5 The Sample tab.

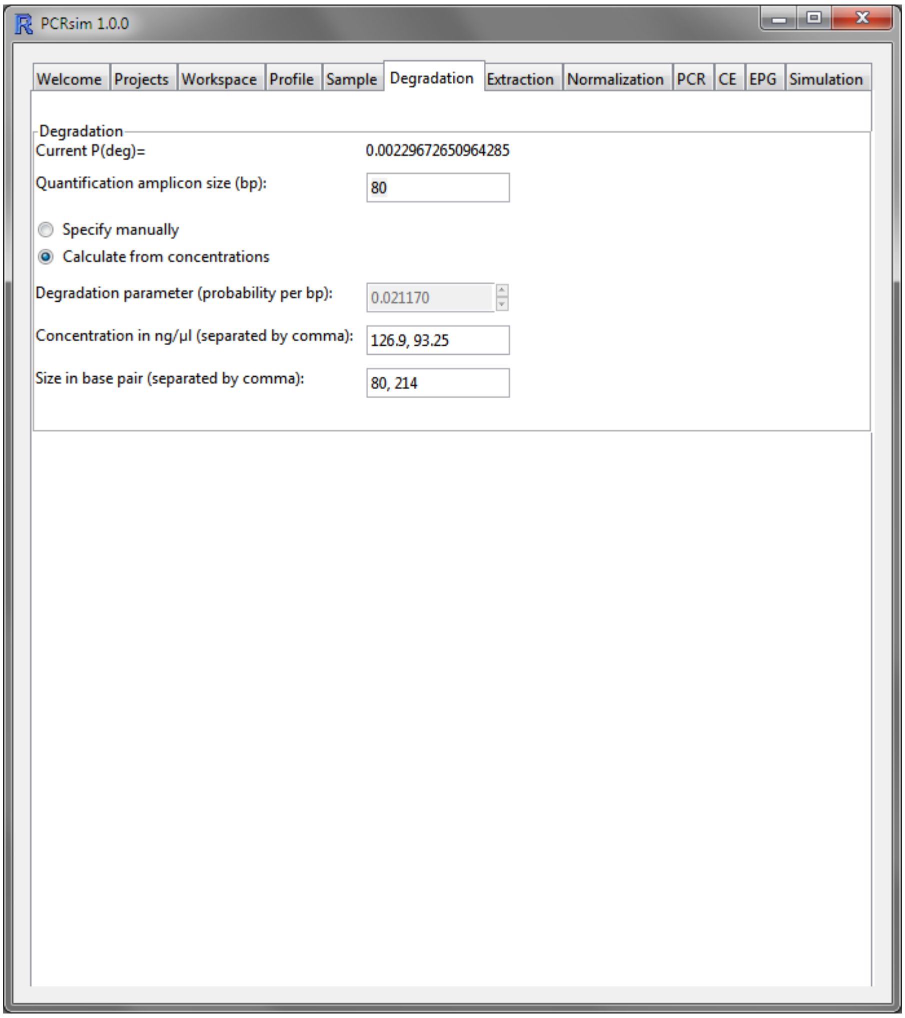

Fig. 6 The Degradation tab.

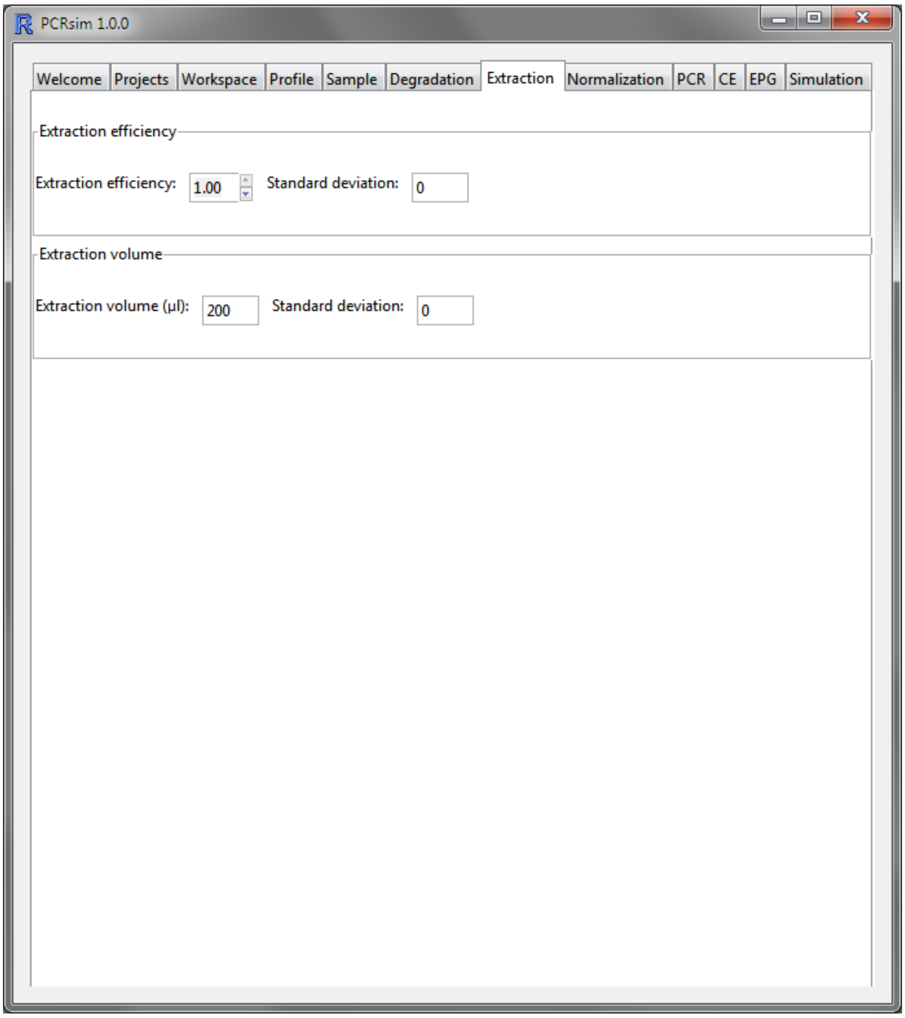

Fig. 7 The Extraction tab.

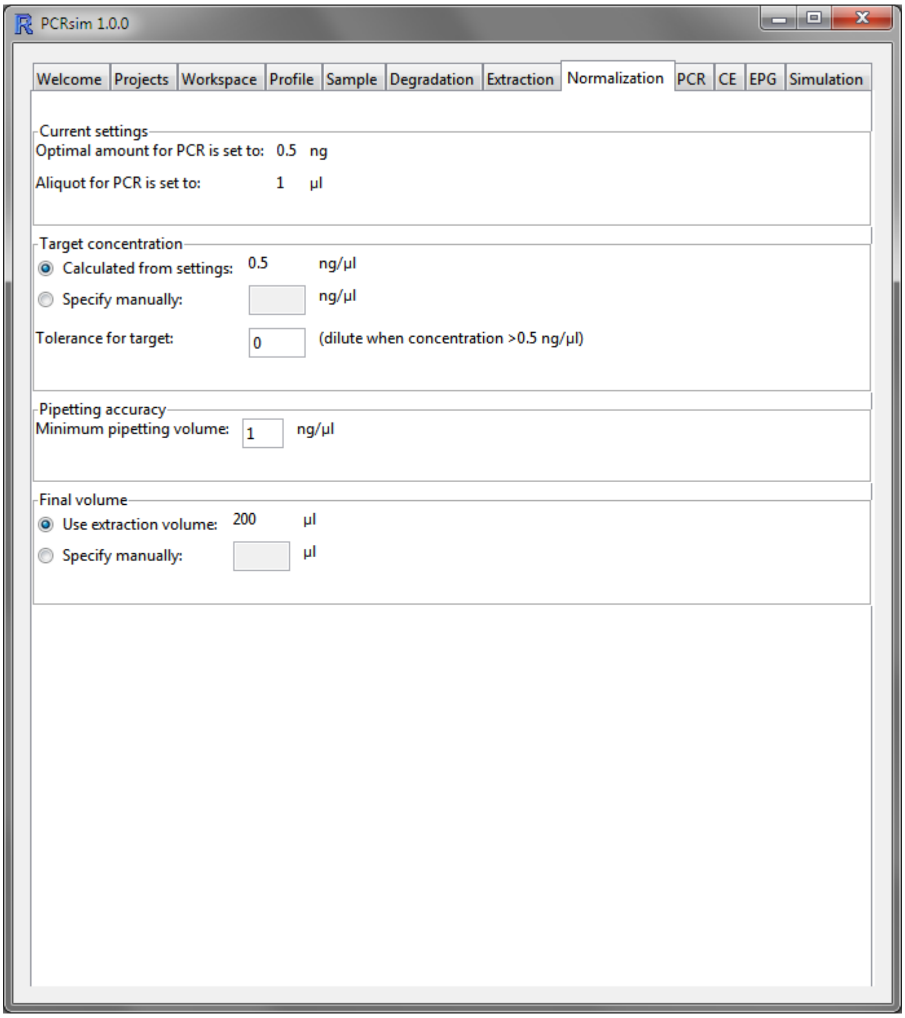

Fig. 8 The Normalization tab.

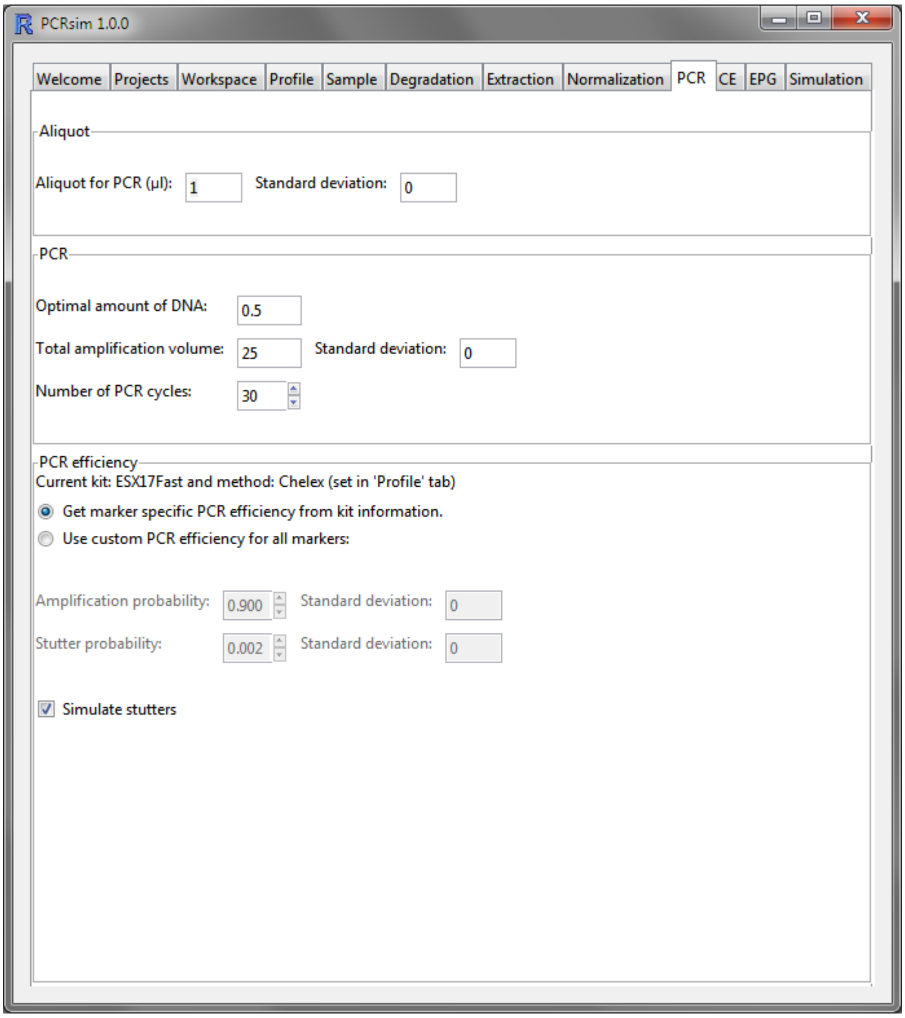

Fig. 9 The PCR tab.

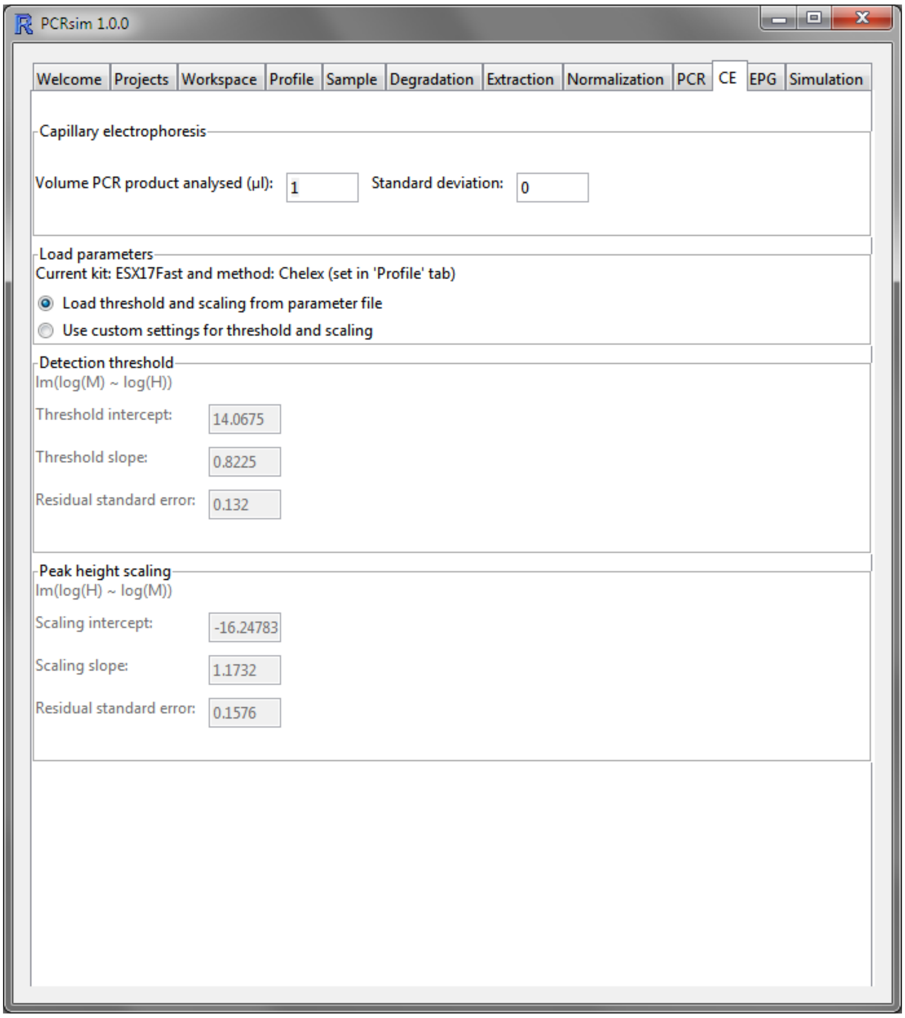

Fig. 10 The CE tab.

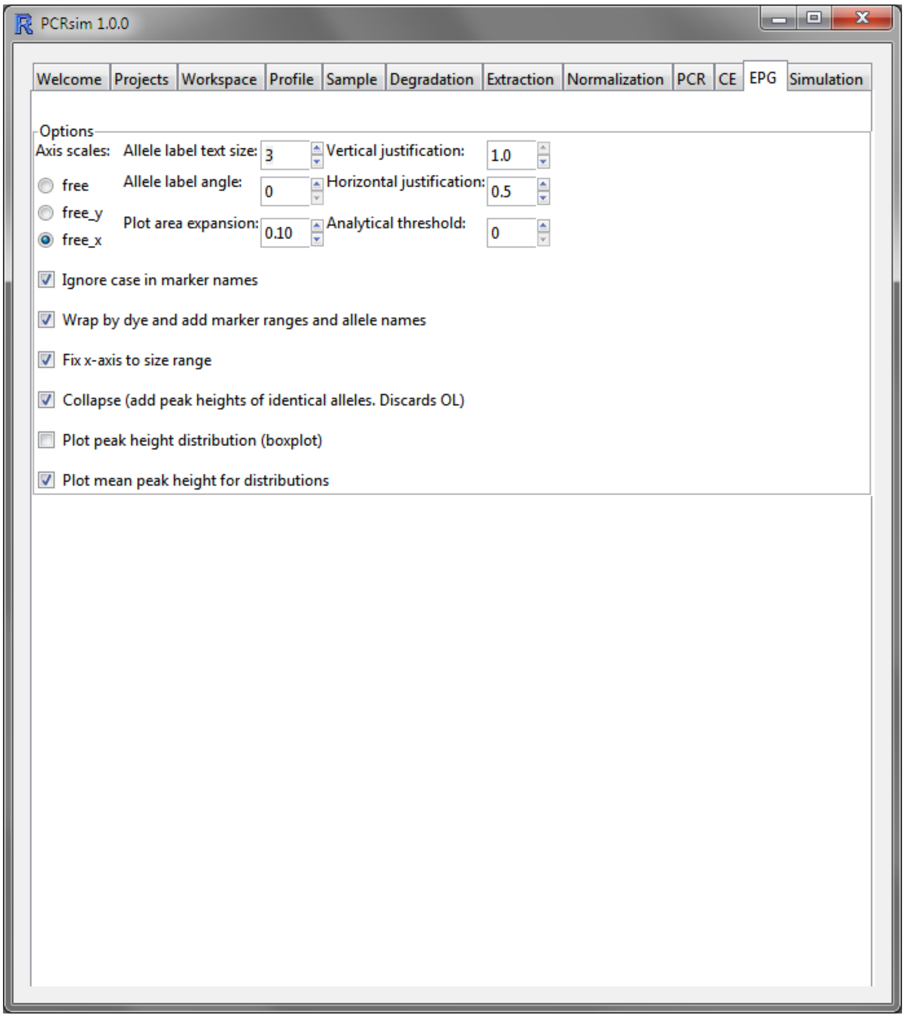

Fig. 11 The EPG tab.

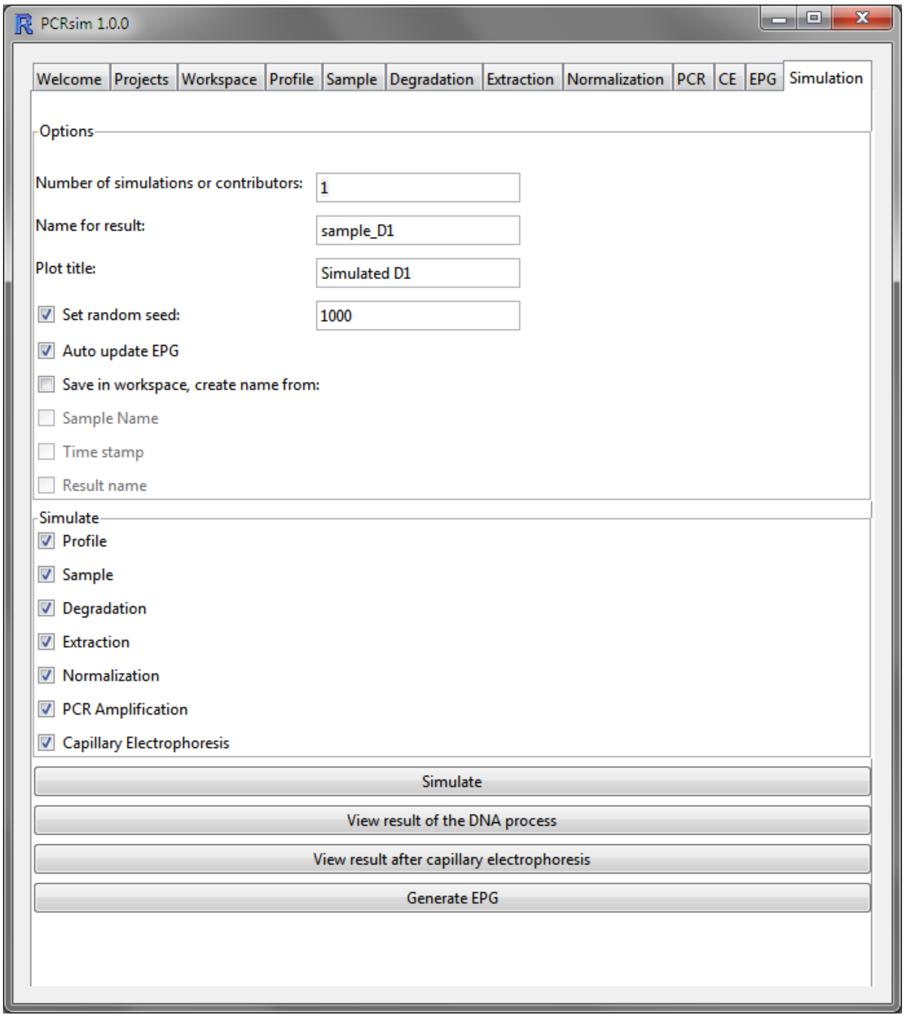

Fig. 12 The Simulation tab.
