# Supplementary material for: Characterization of degradation and heterozygote balance by simulation of the forensic DNA analysis process
Source: Int J Legal Med. 2016 Nov 3;131(2):303–17. doi: 10.1007/s00414-016-1453-x (PMC5306348; doi:10.1007/s00414-016-1453-x)
Supplement: Supplementary file 2 — (ZIP 8.17 MB) [file 414_2016_1453_MOESM2_ESM.zip › ranked_list.pdf]

rank	experiment	amount	sd	n	(est-sim)^2
1	Estimated	84.00	0.2168	1338	0.00E+00
2	Simulated	63.34	0.2148	3793	3.75E-06
3	Simulated	1.22	0.2187	339	3.87E-06
4	Simulated	1.50	0.2132	529	1.24E-05
5	Simulated	59.09	0.2212	3978	2.01E-05
6	Simulated	1.06	0.2101	214	4.40E-05
7	Simulated	0.75	0.2267	91	9.81E-05
8	Simulated	1.31	0.2271	336	1.07E-04
9	Simulated	1.61	0.2284	464	1.36E-04
10	Simulated	67.88	0.2036	3603	1.74E-04

rank	experiment	amount	sd	n	(est-sim)^2
1	Estimated	42.00	0.3233	1219	0.00E+00
2	Simulated	7.39	0.3257	4303	5.31E-06
3	Simulated	6.43	0.3206	3804	7.34E-06
4	Simulated	31.67	0.3159	5643	5.58E-05
5	Simulated	29.55	0.3338	5763	1.09E-04
6	Simulated	7.92	0.3350	4706	1.35E-04
7	Simulated	6.89	0.3108	3993	1.57E-04
8	Simulated	8.49	0.3372	4936	1.93E-04
9	Simulated	27.57	0.3379	5939	2.13E-04
10	Simulated	6.00	0.3081	3761	2.34E-04

rank	experiment	amount	sd	n	(est-sim)^2
1	Estimated	16.80	0.3777	712	0.00E+00
2	Simulated	19.49	0.3747	6426	9.13E-06
3	Simulated	16.97	0.3711	6403	4.33E-05
4	Simulated	18.19	0.3697	6417	6.39E-05
5	Simulated	13.78	0.3684	6232	8.67E-05
6	Simulated	15.83	0.3657	6382	1.45E-04
7	Simulated	12.00	0.3636	6007	2.00E-04
8	Simulated	20.89	0.3631	6359	2.13E-04
9	Simulated	14.77	0.3619	6359	2.50E-04
10	Simulated	22.39	0.3567	6277	4.40E-04

rank	experiment	amount	sd	n	(est-sim)^2
1	Estimated	8.40	0.2477	44	0.00E+00
2	Simulated	2.44	0.2441	1063	1.33E-05
3	Simulated	3.22	0.2516	1511	1.52E-05
4	Simulated	1.40	0.2429	331	2.32E-05
5	Simulated	48.00	0.2527	4584	2.42E-05
6	Simulated	1.98	0.2424	659	2.84E-05
7	Simulated	2.27	0.2412	848	4.30E-05
8	Simulated	1.72	0.2408	508	4.81E-05
9	Simulated	51.45	0.2404	4354	5.41E-05
10	Simulated	2.12	0.2394	851	6.96E-05
