# Supplementary figures and images for: Characterization of degradation and heterozygote balance by simulation of the forensic DNA analysis process
Source: Int J Legal Med. 2016 Nov 3;131(2):303–17. doi: 10.1007/s00414-016-1453-x (PMC5306348; doi:10.1007/s00414-016-1453-x)

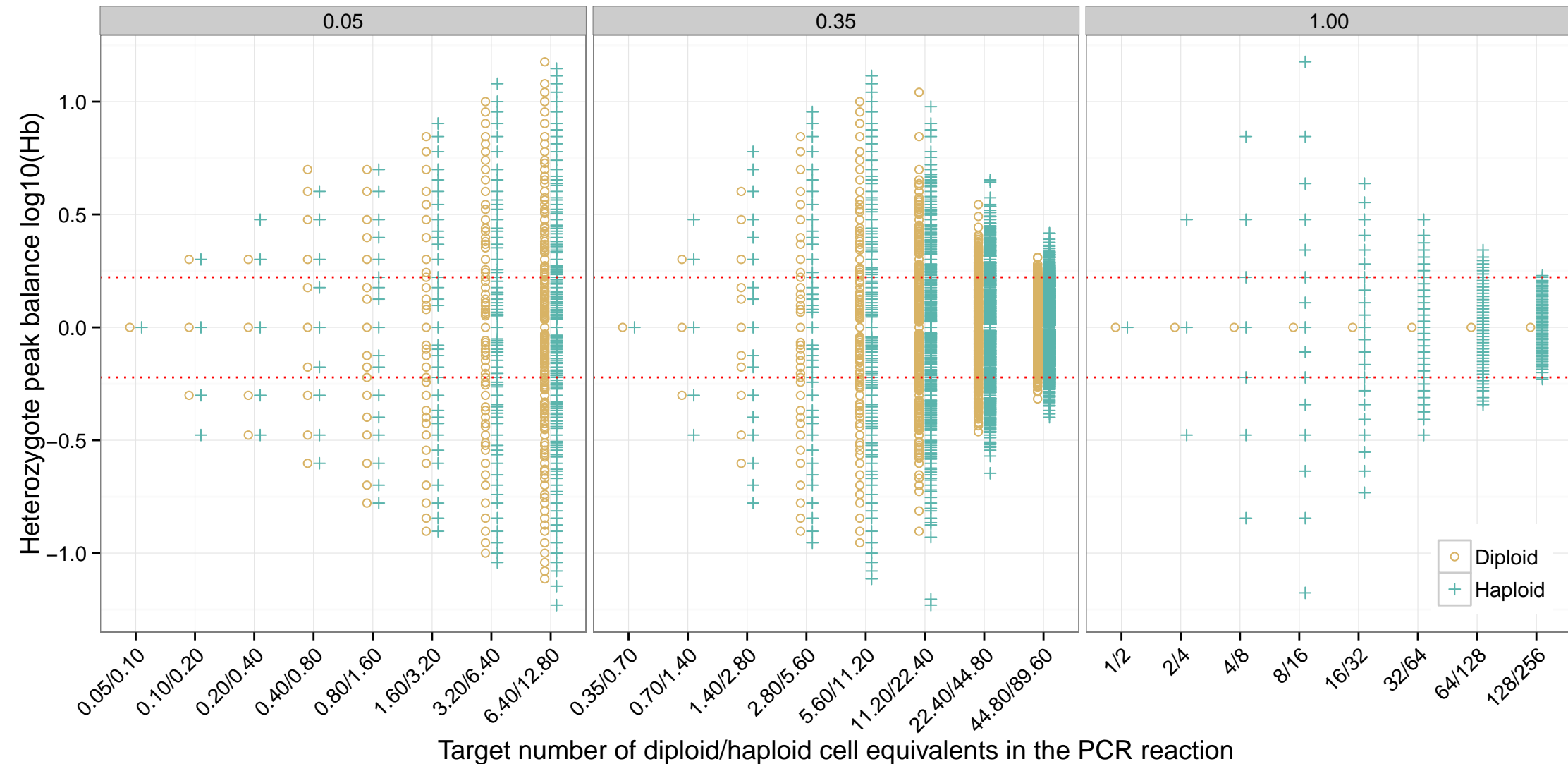

Supplement: Supplementary file 2 — (ZIP 8.17 MB) [file 414_2016_1453_MOESM2_ESM.zip › aliquot_proportion_1500.pdf]

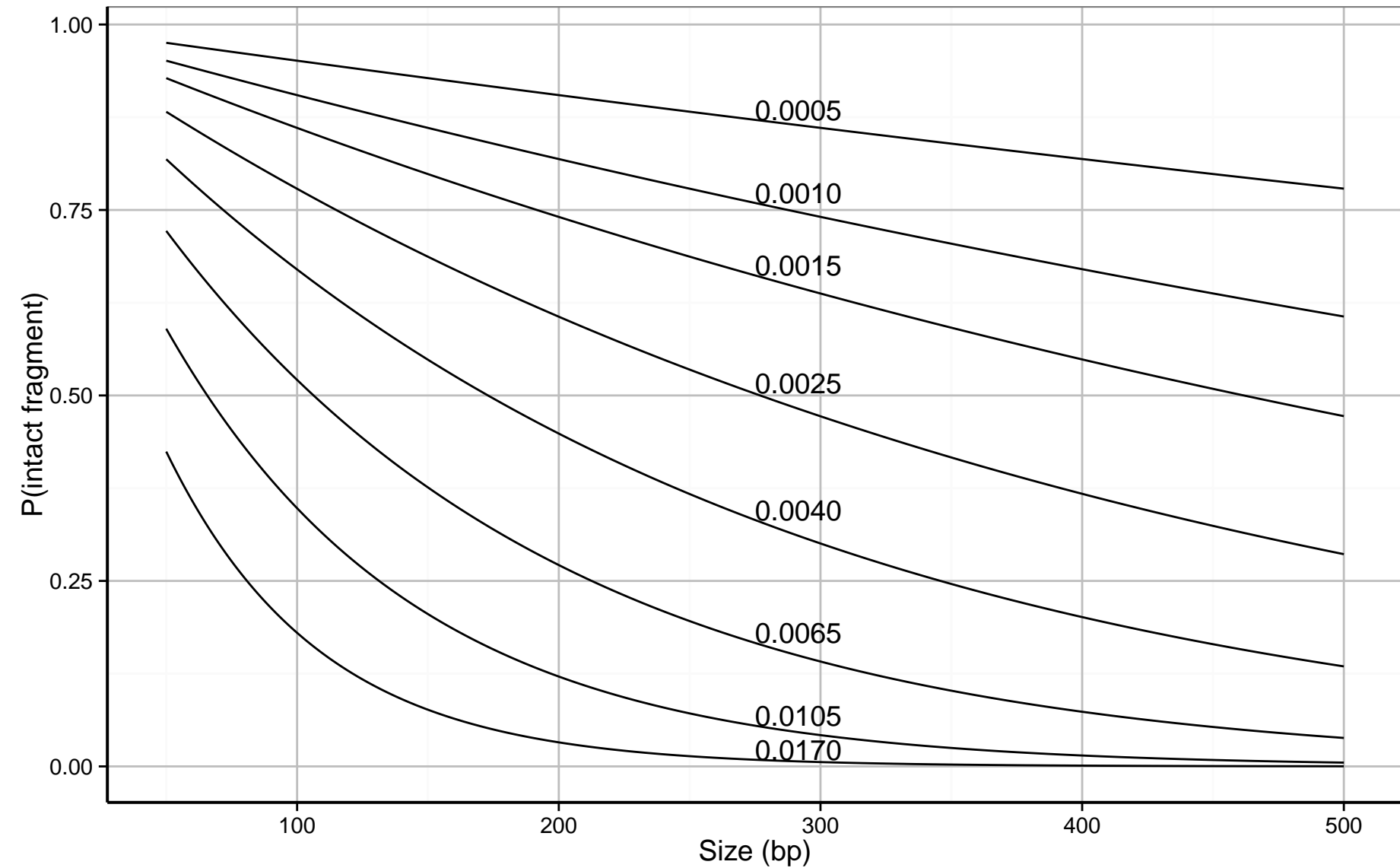

Supplement: Supplementary file 2 — (ZIP 8.17 MB) [file 414_2016_1453_MOESM2_ESM.zip › deg_grid.pdf]

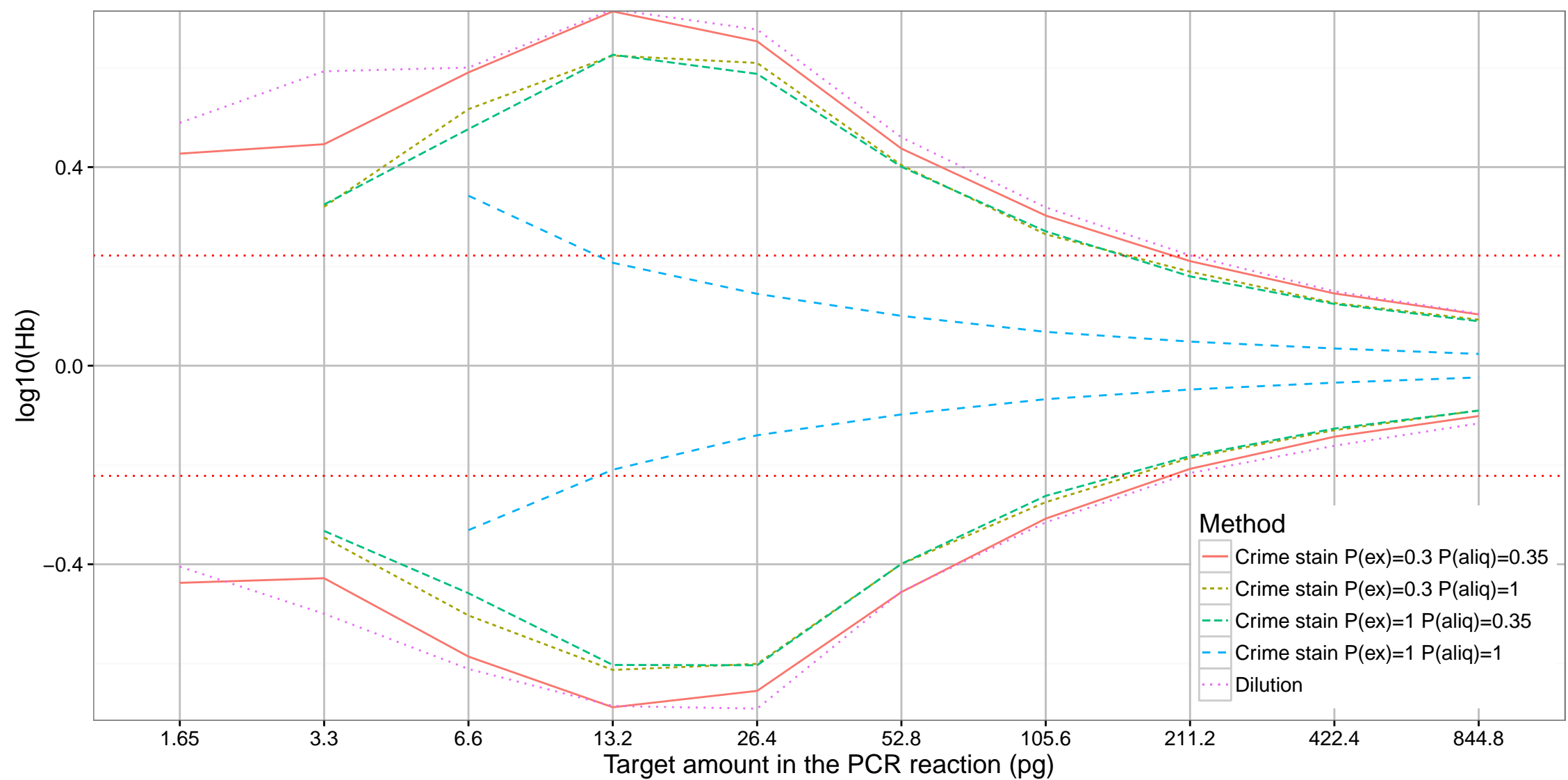

Supplement: Supplementary file 2 — (ZIP 8.17 MB) [file 414_2016_1453_MOESM2_ESM.zip › dilution_vs_stain_diploid_1000_90.pdf]

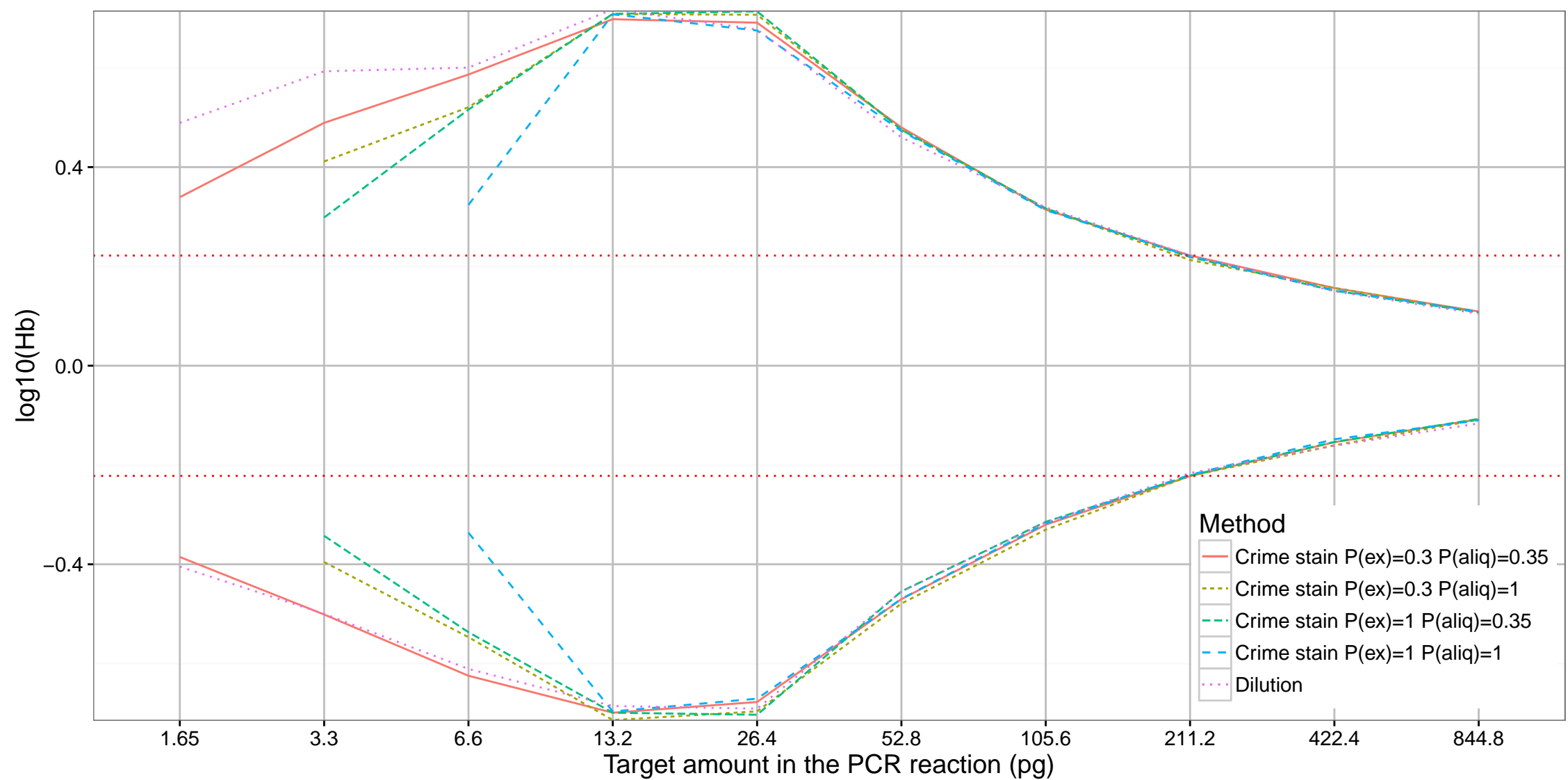

Supplement: Supplementary file 2 — (ZIP 8.17 MB) [file 414_2016_1453_MOESM2_ESM.zip › dilution_vs_stain_haploid_1000_90.pdf]

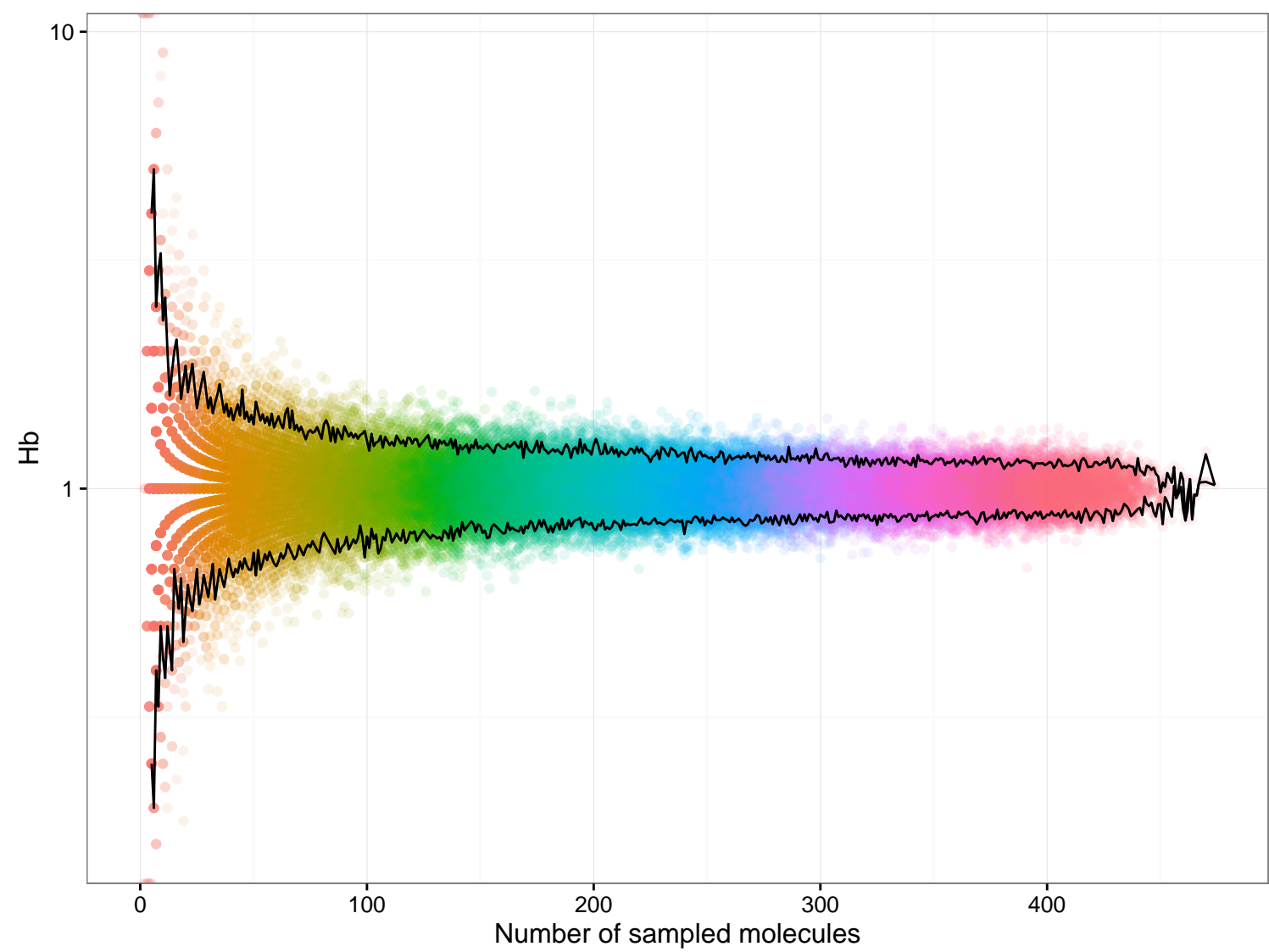

Supplement: Supplementary file 2 — (ZIP 8.17 MB) [file 414_2016_1453_MOESM2_ESM.zip › discrete_possibilities_35.pdf]

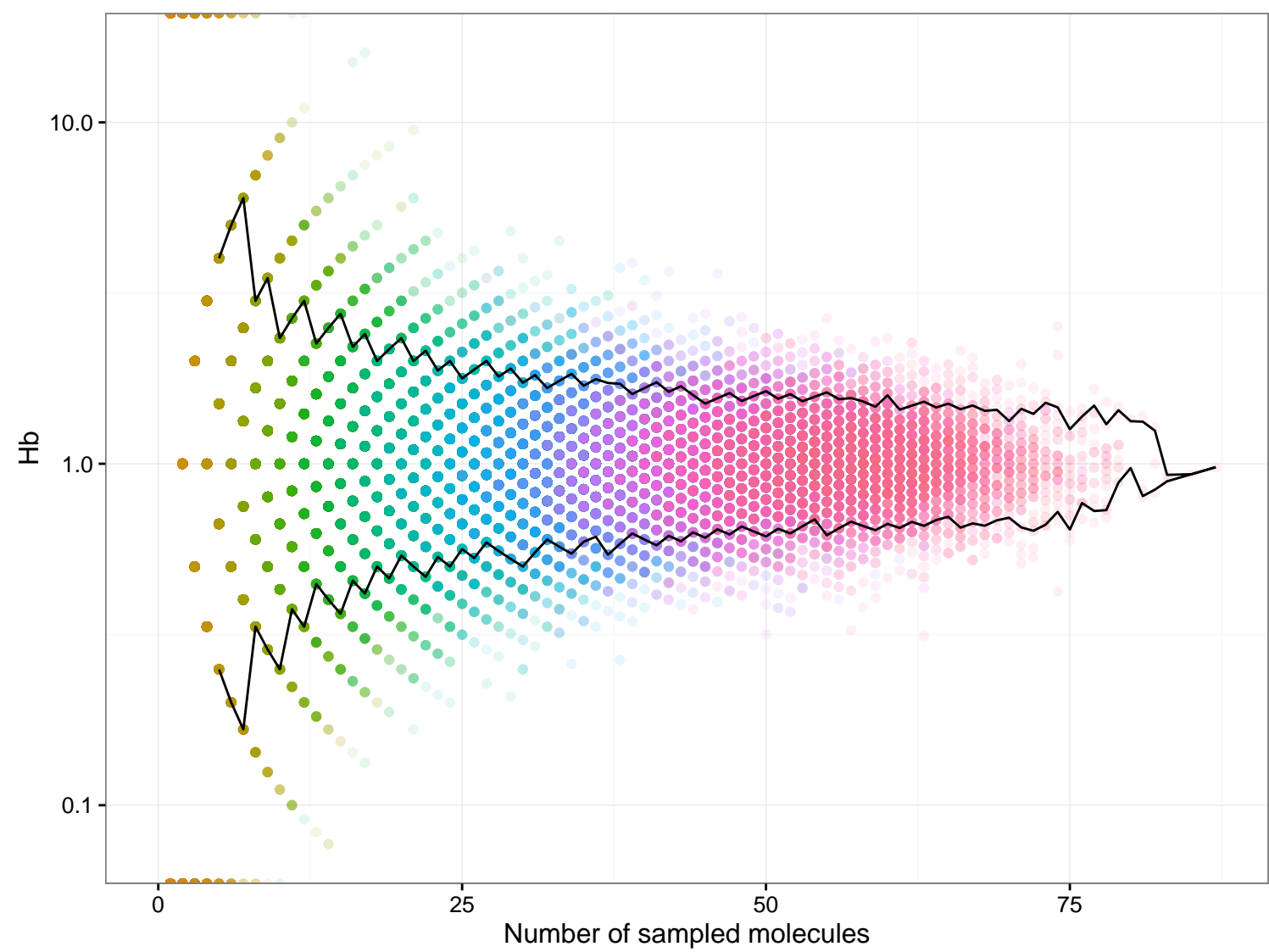

Supplement: Supplementary file 2 — (ZIP 8.17 MB) [file 414_2016_1453_MOESM2_ESM.zip › discrete_possibilities_5.pdf]

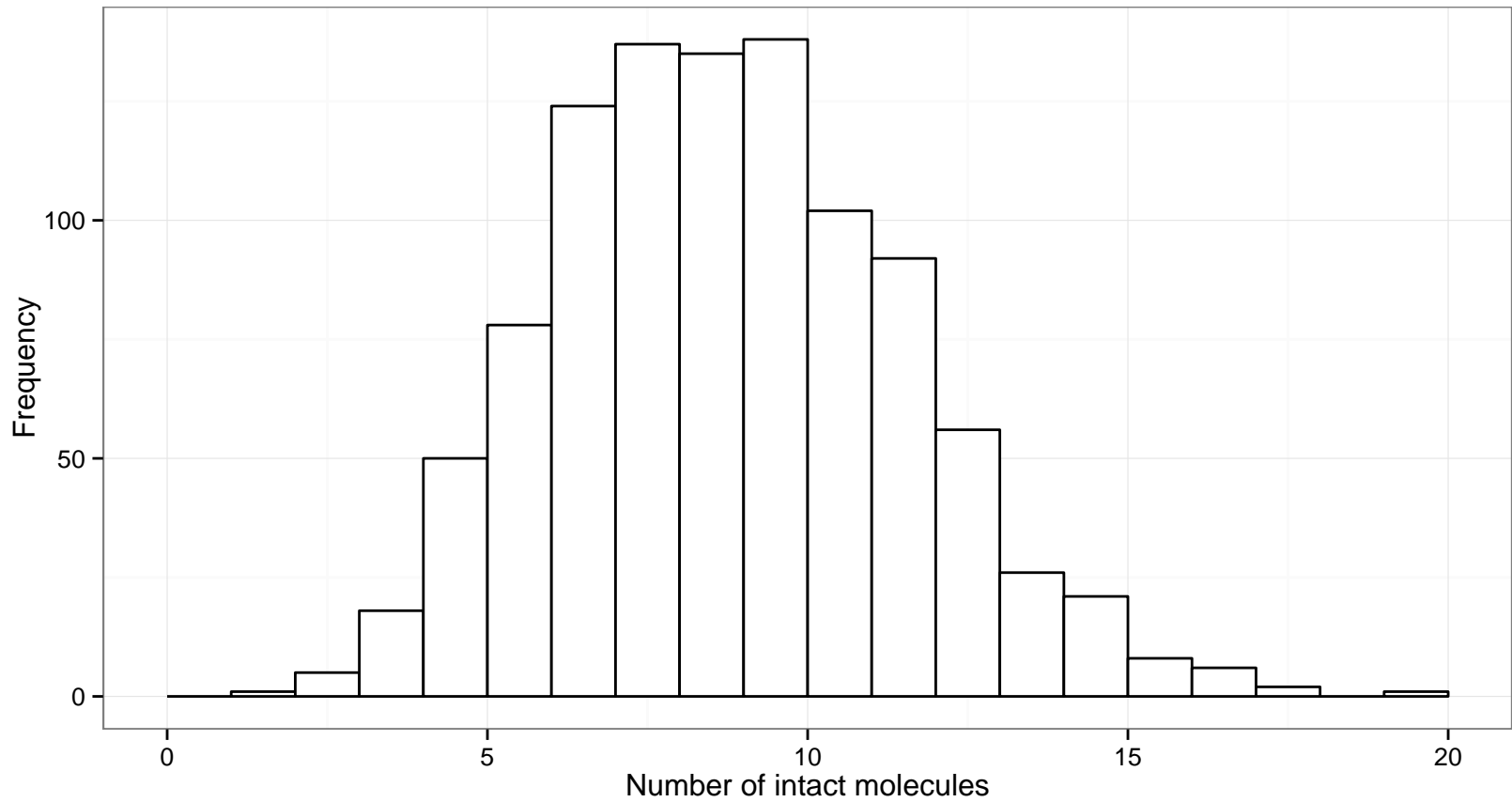

Supplement: Supplementary file 2 — (ZIP 8.17 MB) [file 414_2016_1453_MOESM2_ESM.zip › example_intact_molecules_long.pdf]

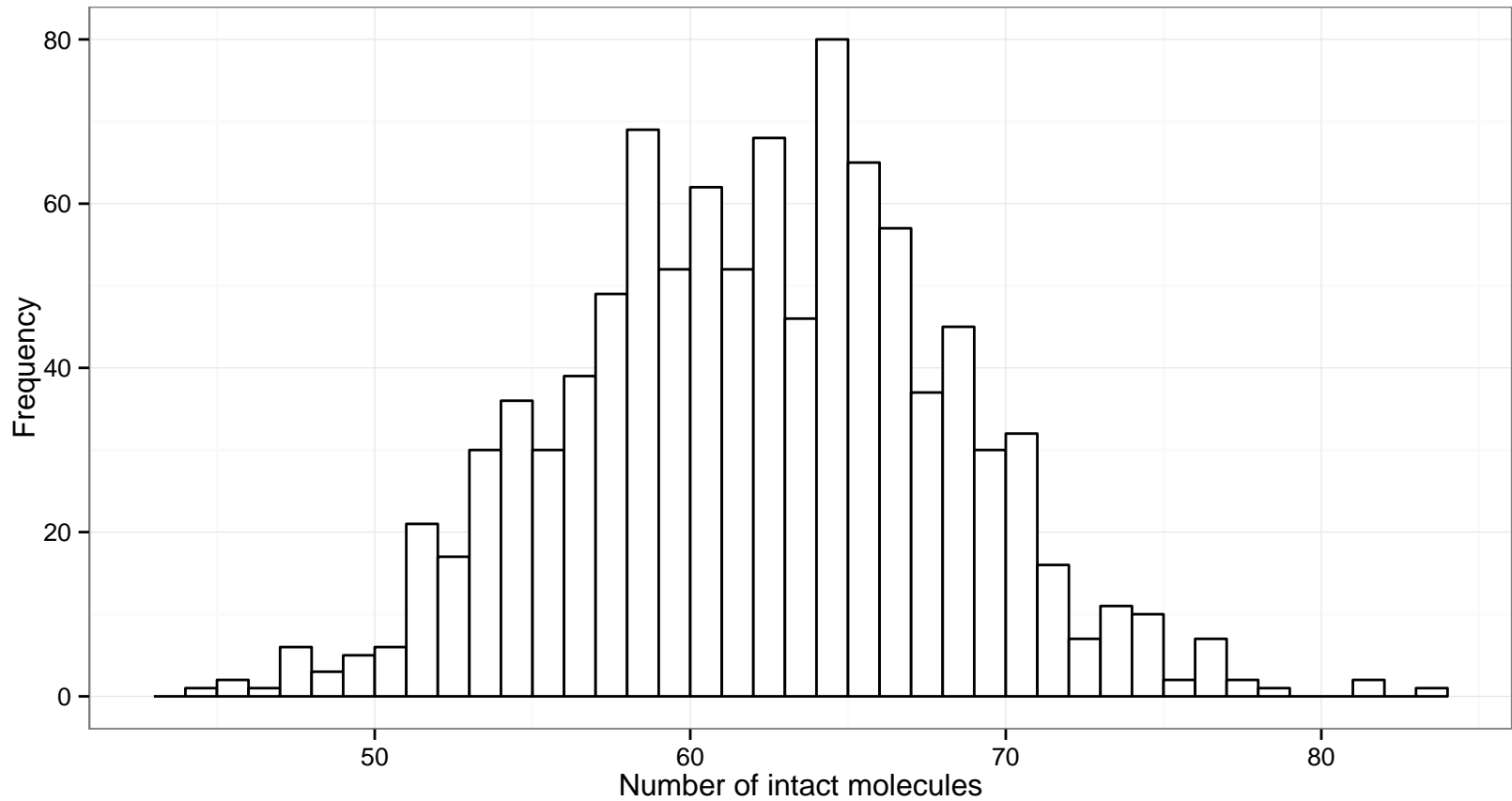

Supplement: Supplementary file 2 — (ZIP 8.17 MB) [file 414_2016_1453_MOESM2_ESM.zip › example_intact_molecules_short.pdf]

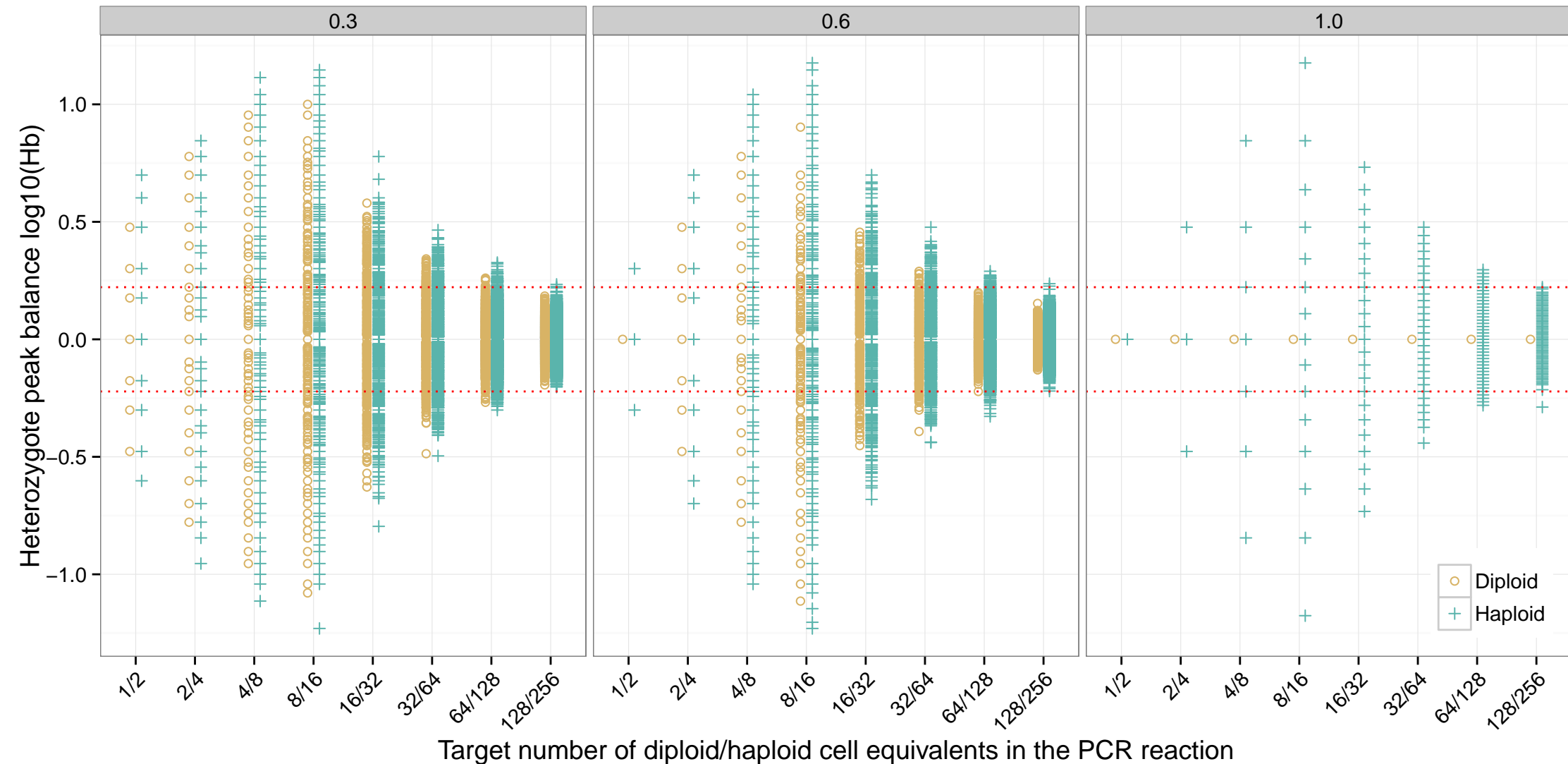

Supplement: Supplementary file 2 — (ZIP 8.17 MB) [file 414_2016_1453_MOESM2_ESM.zip › extraction_efficiency_1000.pdf]

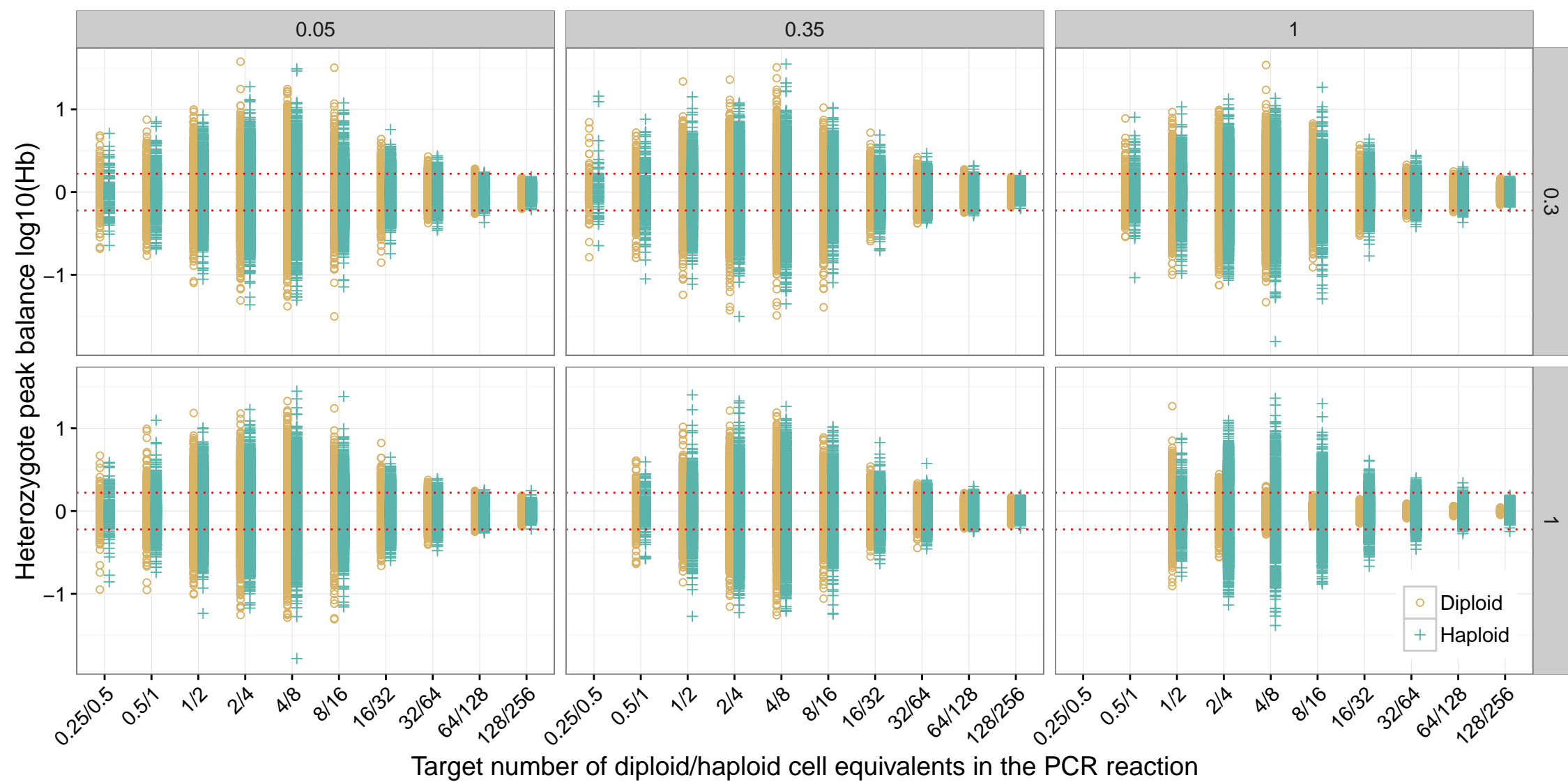

Supplement: Supplementary file 2 — (ZIP 8.17 MB) [file 414_2016_1453_MOESM2_ESM.zip › extraction_vs_aliquot.pdf]

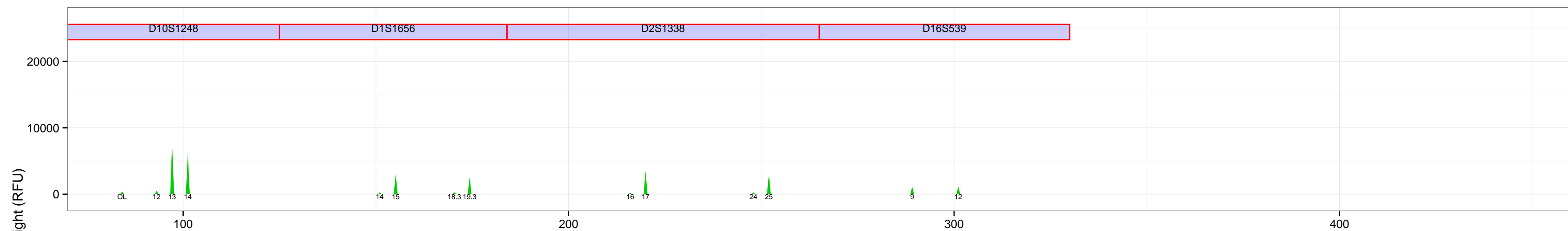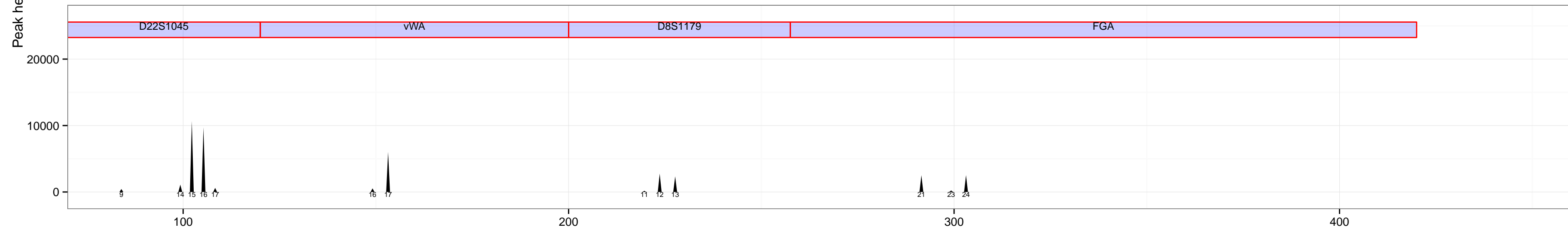

Supplement: Supplementary file 2 — (ZIP 8.17 MB) [file 414_2016_1453_MOESM2_ESM.zip › observed_green_yellow.pdf]

Heterozygote peak balance  $\log_{10}(\text{Hb})$

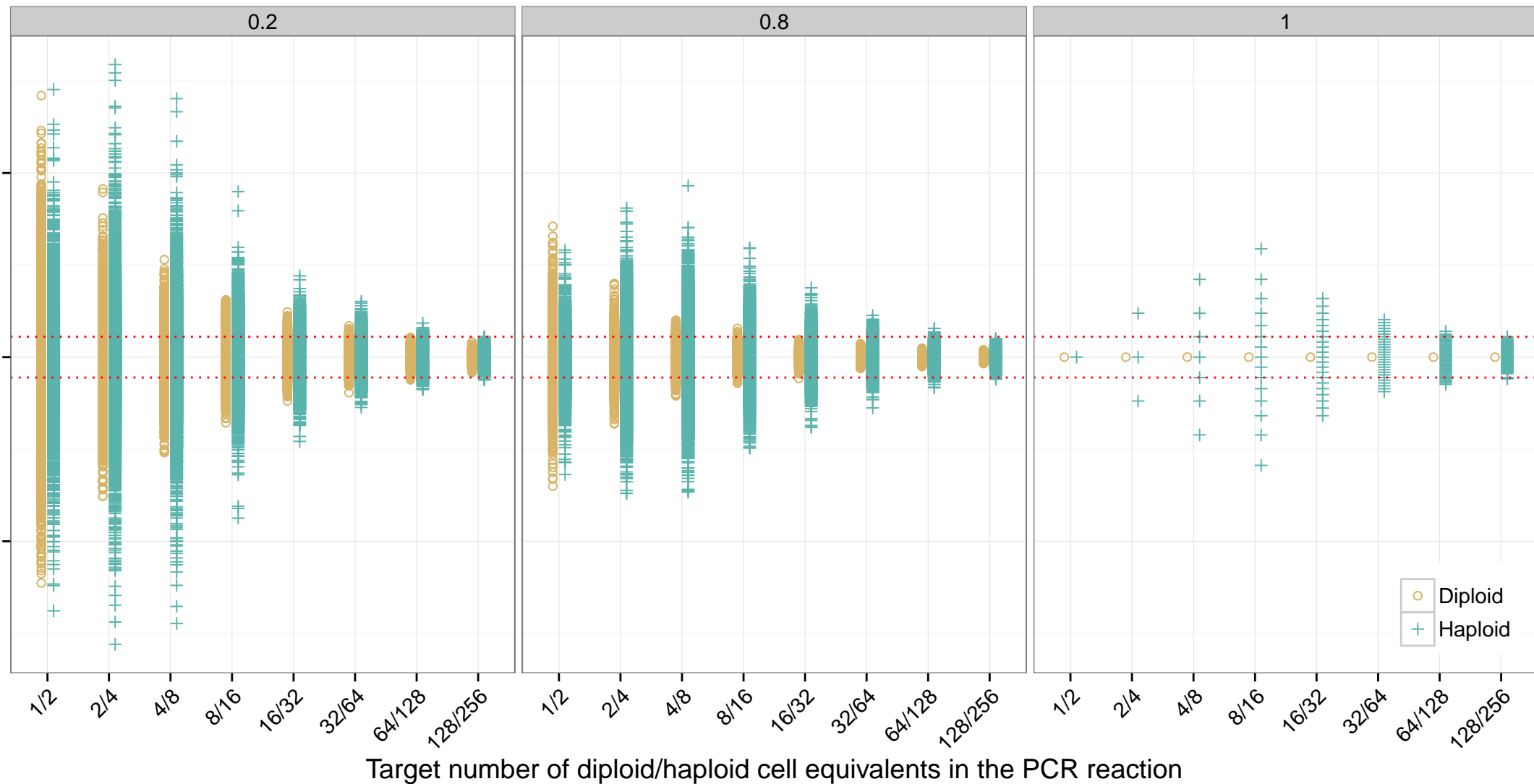

Supplement: Supplementary file 2 — (ZIP 8.17 MB) [file 414_2016_1453_MOESM2_ESM.zip › pcr_efficiency_500.pdf]

Proportion

1.00 -  
0.75 -  
0.50 -  
0.25 -  
0.00 -

0

25

50

75

100

Number of molecules in extract

Alleles

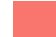

one

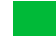

both

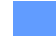

none

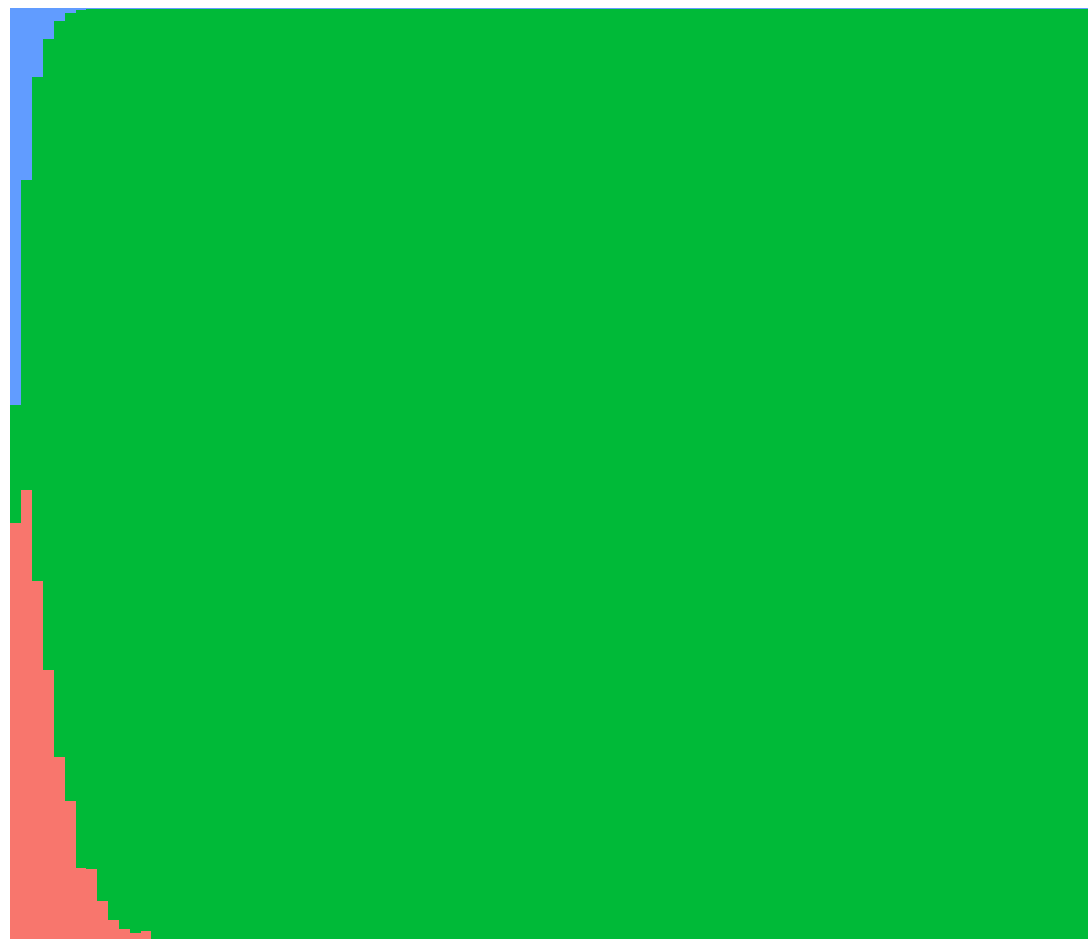

Supplement: Supplementary file 2 — (ZIP 8.17 MB) [file 414_2016_1453_MOESM2_ESM.zip › sampled_alleles_35.pdf]

Proportion

1.00 -  
0.75 -  
0.50 -  
0.25 -  
0.00 -

0

25

50

75

100

Number of molecules in extract

Alleles

one

both

none

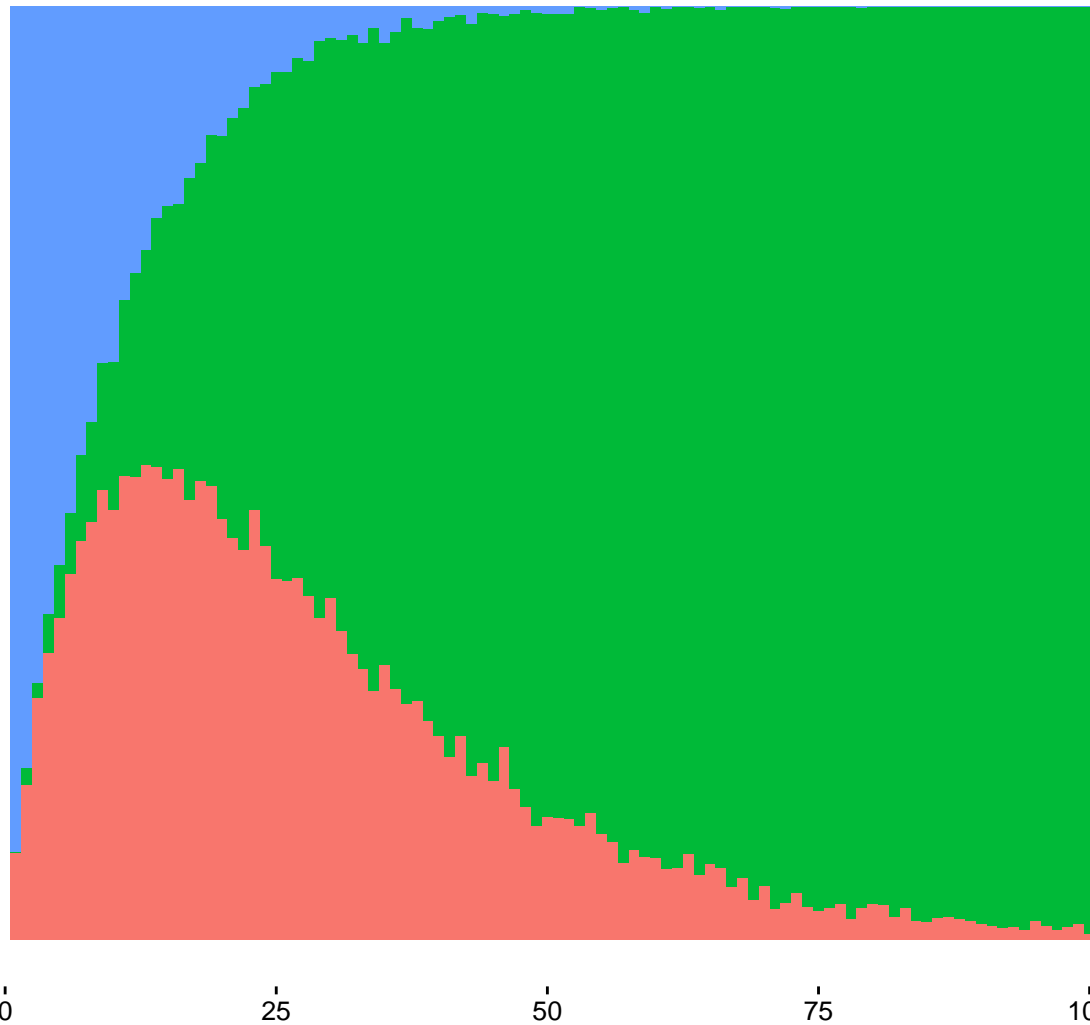

Supplement: Supplementary file 2 — (ZIP 8.17 MB) [file 414_2016_1453_MOESM2_ESM.zip › sampled_alleles_5.pdf]

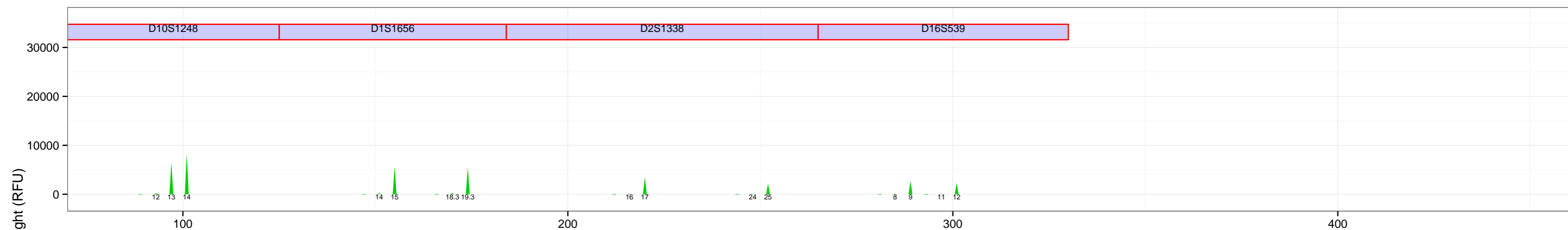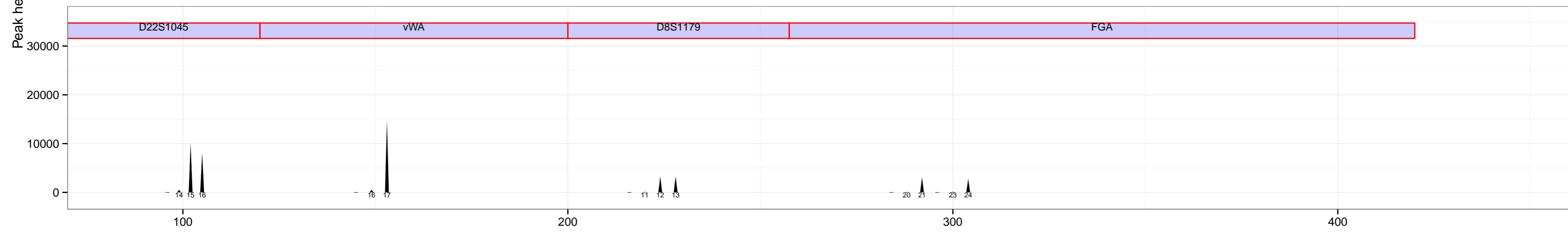

Supplement: Supplementary file 2 — (ZIP 8.17 MB) [file 414_2016_1453_MOESM2_ESM.zip › simulated_green_yellow.pdf]

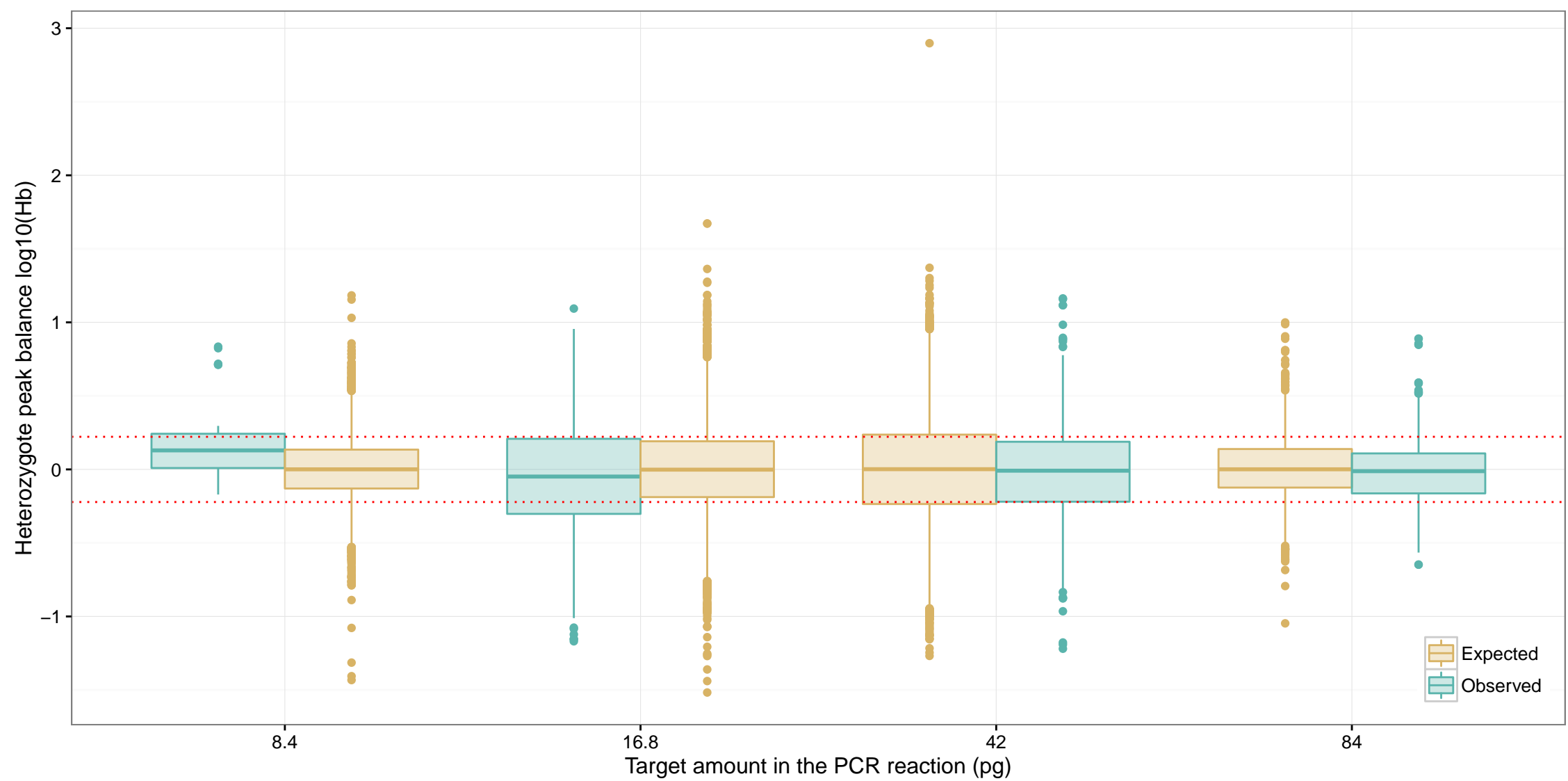

Supplement: Supplementary file 2 — (ZIP 8.17 MB) [file 414_2016_1453_MOESM2_ESM.zip › skl_vs_sim.pdf]

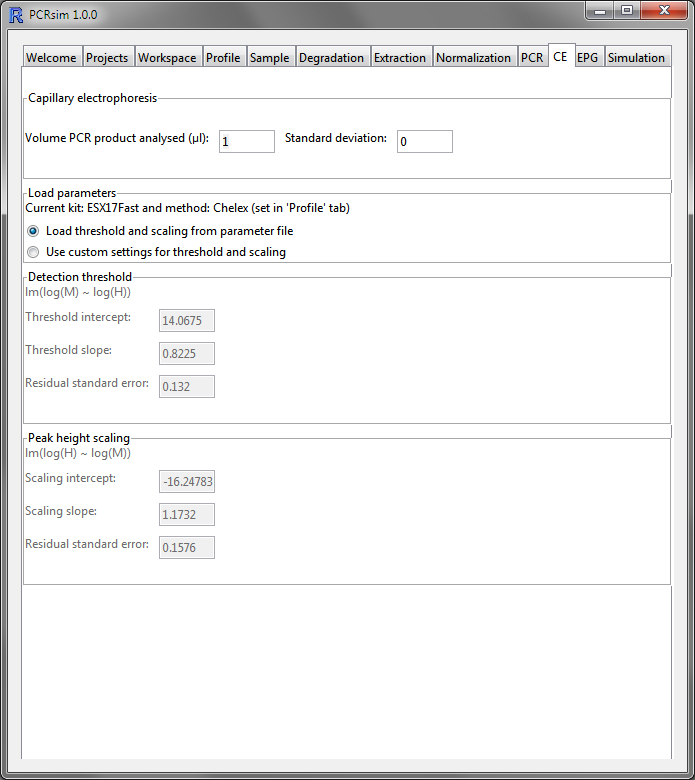

Supplement: Supplementary file 2 — (ZIP 8.17 MB) [file 414_2016_1453_MOESM2_ESM.zip › tab_ce.png]

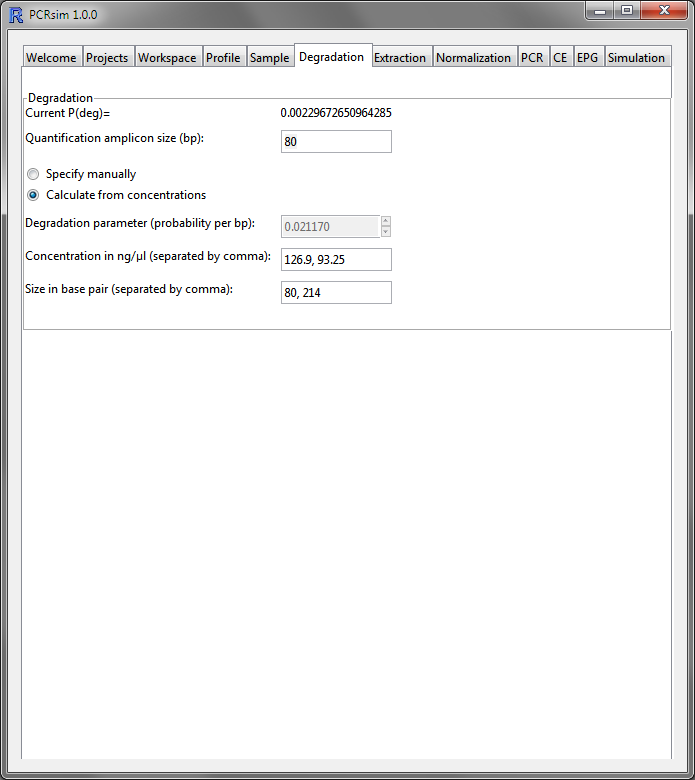

Supplement: Supplementary file 2 — (ZIP 8.17 MB) [file 414_2016_1453_MOESM2_ESM.zip › tab_degradation.png]

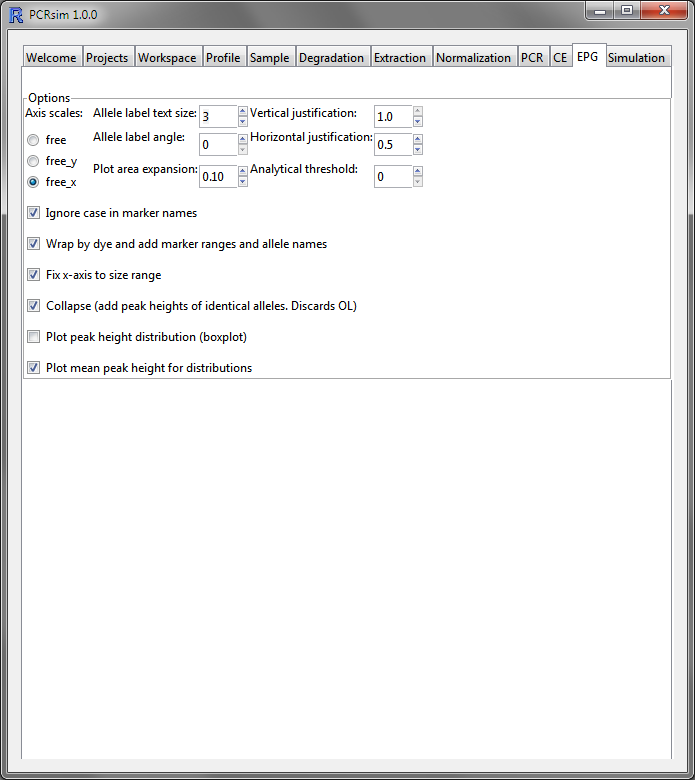

Supplement: Supplementary file 2 — (ZIP 8.17 MB) [file 414_2016_1453_MOESM2_ESM.zip › tab_epg.png]

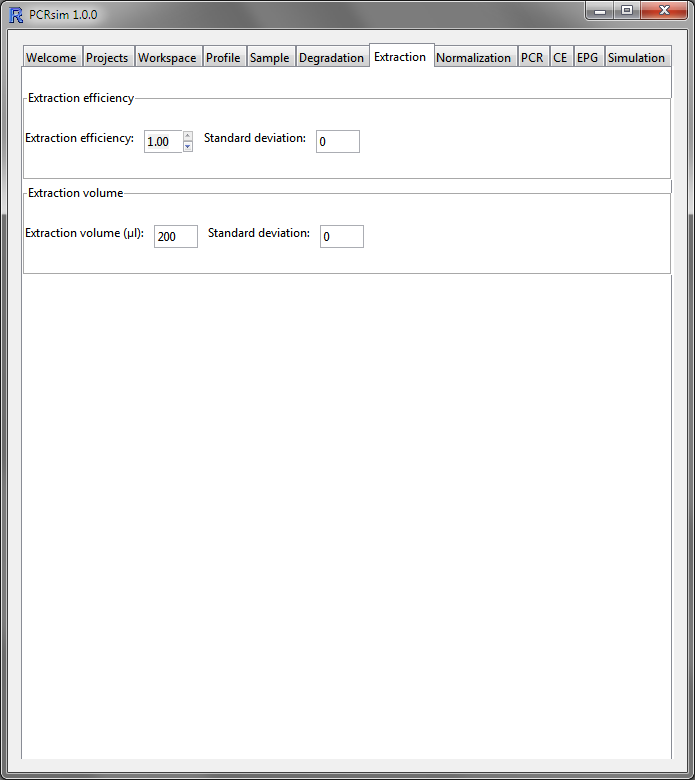

Supplement: Supplementary file 2 — (ZIP 8.17 MB) [file 414_2016_1453_MOESM2_ESM.zip › tab_extraction.png]

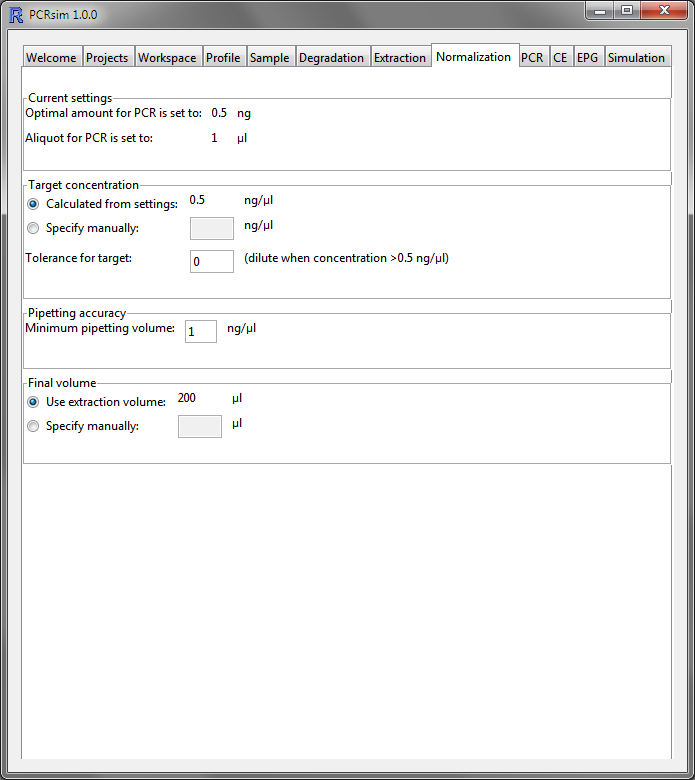

Supplement: Supplementary file 2 — (ZIP 8.17 MB) [file 414_2016_1453_MOESM2_ESM.zip › tab_normalization.png]

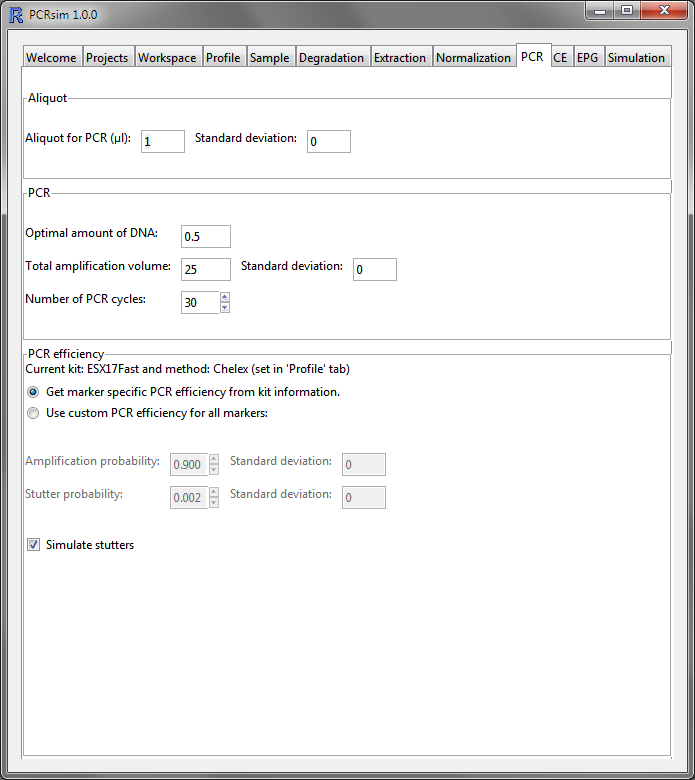

Supplement: Supplementary file 2 — (ZIP 8.17 MB) [file 414_2016_1453_MOESM2_ESM.zip › tab_pcr.png]

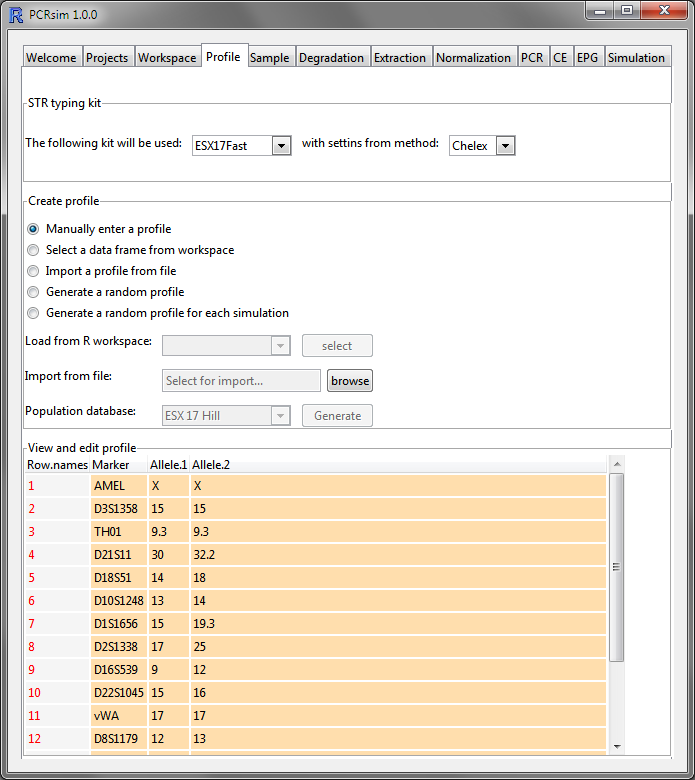

Supplement: Supplementary file 2 — (ZIP 8.17 MB) [file 414_2016_1453_MOESM2_ESM.zip › tab_profile.png]

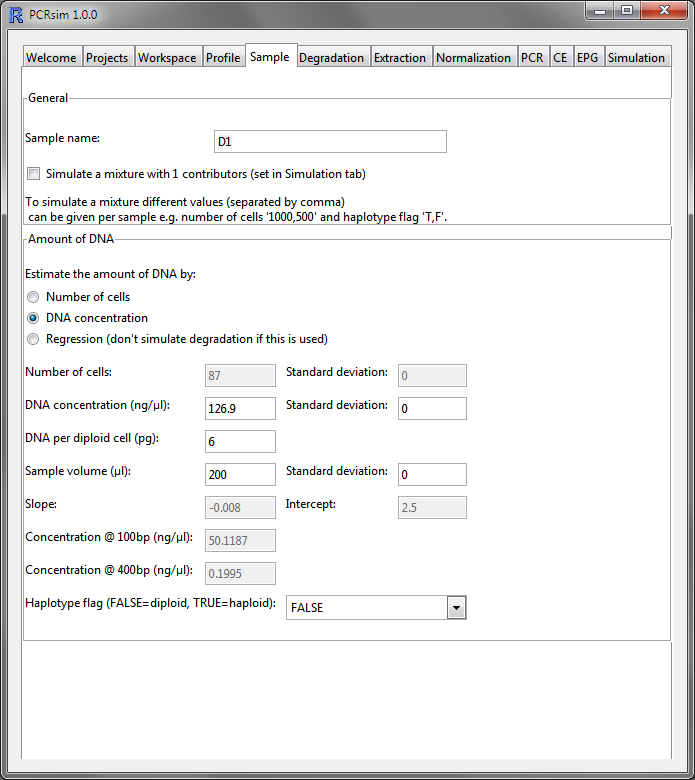

Supplement: Supplementary file 2 — (ZIP 8.17 MB) [file 414_2016_1453_MOESM2_ESM.zip › tab_sample.png]

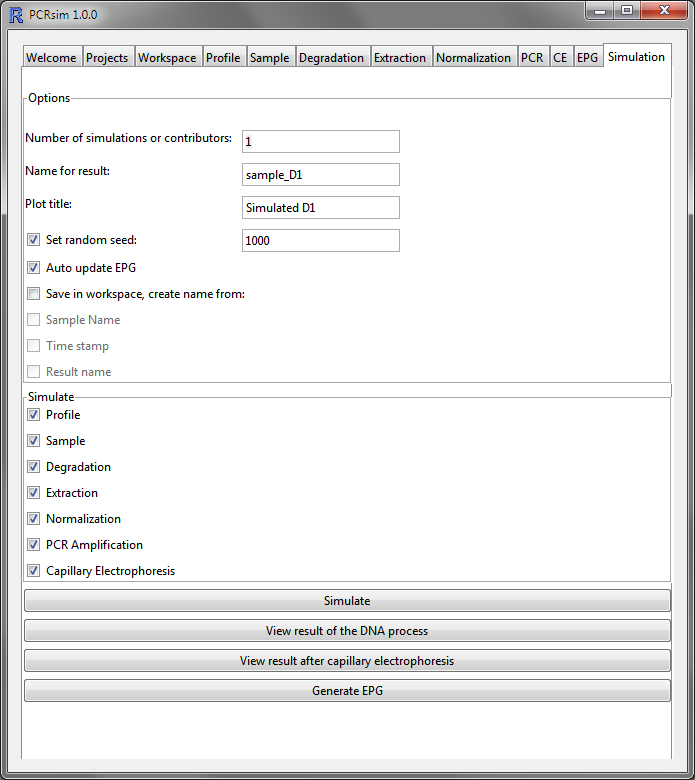

Supplement: Supplementary file 2 — (ZIP 8.17 MB) [file 414_2016_1453_MOESM2_ESM.zip › tab_simulation.png]
